# How Humans Solve Complex Problems: The Case of the Knapsack Problem

**DOI:** 10.1038/srep34851

**Published:** 2016-10-07

**Authors:** Carsten Murawski, Peter Bossaerts

**Affiliations:** 1 Department of Finance, The University of Melbourne, Parkville, Victoria 3010, Australia; 2 The Florey Institute of Neuroscience and Mental Health, Parkville, Victoria 3010, Australia

## Abstract

Life presents us with problems of varying complexity. Yet, complexity is not accounted for in theories of human decision-making. Here we study instances of the knapsack problem, a discrete optimisation problem commonly encountered at all levels of cognition, from attention gating to intellectual discovery. Complexity of this problem is well understood from the perspective of a mechanical device like a computer. We show experimentally that human performance too decreased with complexity as defined in computer science. Defying traditional economic principles, participants spent effort way beyond the point where marginal gain was positive, and economic performance increased with instance difficulty. Human attempts at solving the instances exhibited commonalities with algorithms developed for computers, although biological resource constraints–limited working and episodic memories–had noticeable impact. Consistent with the very nature of the knapsack problem, only a minority of participants found the solution–often quickly–but the ones who did appeared not to realise. Substantial heterogeneity emerged, suggesting why prizes and patents, schemes that incentivise intellectual discovery but discourage information sharing, have been found to be less effective than mechanisms that reveal private information, such as markets.

The knapsack problem (KP) is a combinatorial optimisation problem with the goal of finding, in a set of items of given values and weights, the subset of items with the highest total value, subject to a total weight constraint^1^,^2^ (Supplementary Methods 1.1). It is a member of the complexity class *non-deterministic polynomial-time (NP) hard*^2^,^3^,^4^. For those problems, there are no known efficient solution algorithms, that is, algorithms whose computational time only grows as a polynomial of the size of the problem’s instances^5^. This feature obtains despite the fact that one can compute relatively fast whether a given candidate solution reaches a certain value level.

The KP permeates the lives of humans (and non-human animals). It emerges at a low level of cognition, for example in the choice of stimuli to attend to (visual, auditory, tactile). At an intermediate level of cognition, the KP can be recognised in tasks like budgeting and time management^6^. At the highest level, it occurs for example in production (cutting problems^7^), logistics (bin packing problems^8^), combinatorial auctions, and, in financial economics, in portfolio optimisation^9^. It has also been argued that the KP reflects an important aspect of innovation and intellectual discovery^10^,^11^,^12^,^13^,^14^. Consistent with this view, a recent empirical study showed that most patents filed at the U.S. Patent Office between 1790 and 2010 were for inventions that combined existing technologies in novel ways^14^. Furthermore, the KP is related to mental disorders. For example, several of the symptoms of attention-deficit/hyperactivity disorder can be regarded as impaired ability to solve the KP^6^.

Here, we examine whether the degree of complexity of a computational problem affects human behaviour, and if so, what strategies humans resort to. Twenty healthy participants attempted to solve eight instances of the 0–1 knapsack problem^12^, administered on a computer (Fig. 1a–b, Supplementary Methods 1.6). In each instance, participants were presented with a set of items of differing values and weights (Table S1). Participants used a mouse to add available items to, and remove items from, the knapsack. Total value and weight of the selected items were displayed at the top of the screen. Participants were offered two attempts per instance. The time limit per attempt was four minutes. Participants were asked to press the space bar when they believed that they had found the solution of an instance, to submit the solution. Submitted attempts were rewarded based on an economic performance measure that increased in closeness in values between the solution submitted (“attained value”) and the optimal knapsack (“optimal solution value”).

## Results

### Performance

Computational performance, defined as the proportion of attempts (“score”) of an instance in which participants found the maximum value, was 37.4%. The mean time on task at submission was 172 s (SD = 57 s), and 97.5% of attempts were submitted before the time limit of four minutes. While computational performance indicates that our instances were difficult indeed, there was substantial variability across instances (min = 0.027, M = 0.367, max = 0.744, SD = 0.19; Fig. 2a), and across participants (min = 0.062, M = 0.374, max = 0.562, SD = 0.157; Fig. 2b). Computational performance was significantly higher than what participants would have achieved by implementing stochastic search (‘trial and error’). Participants on average submitted only 14 different sets of items (SD = 7). In comparison, the average number of possible configurations of full (capacity-constrained) knapsacks in the different instances was 1,381. The chance that any capacity-constrained knapsack was the optimal one was a mere 0.7%, implying an expected computational performance from random search equal to only 0.10 (SD = 0.05). The total number of correct attempts was significantly above chance (*P < *0.001, one-sided binomial test). This suggests that searches were systematic, that is, based on a non-random protocol.

Economic performance was defined as the value attained in the submitted solution as a percentage of optimal solution value. Participant earnings were based on economic performance (Supplementary Methods 1.6). We found that, on average, participants submitted solutions with value equal to 97.4% of optimal solution value. This value was significantly higher than the expected economic performance of an algorithm that randomly picks a capacity-constrained knapsack, which was 85.3% (*P < *0.001, one-sample t-test, *t*(307) = 36.382). Similar to computational performance, economic performance varied more by instance (min = 95.8%, M = 97.4%, max = 99.0%, SD = 1.1%) than by participant (min = 88.9%, M = 97.4%, max = 99.3%, SD = 2.4%).

### Effort

**Figure 1:**
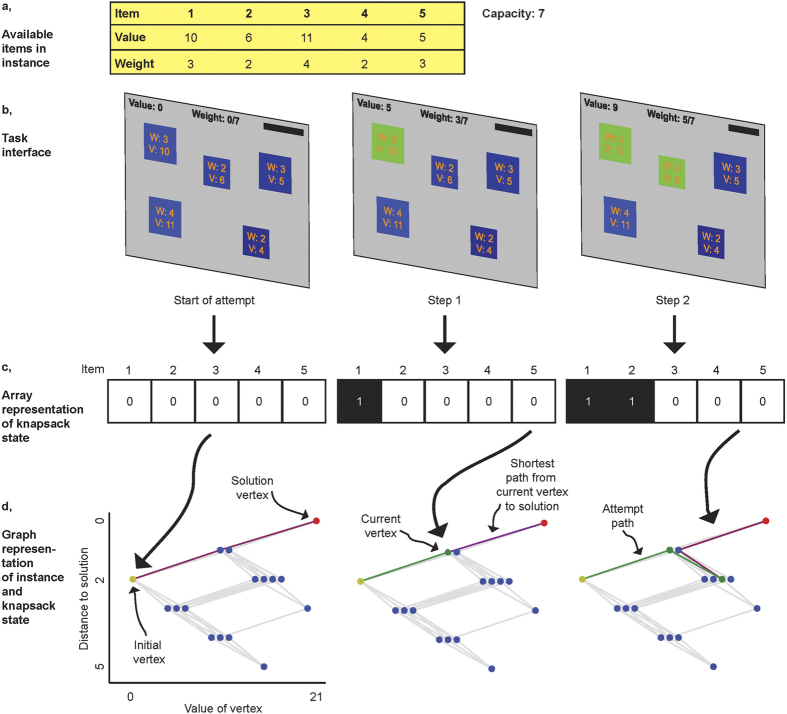
Overview of experimental paradigm. (**a**) Example instance of the 0–1 knapsack problem with five items. The goal is to find the subset of available items that maximises total value of the knapsack, subject to a capacity constraint. Values and weights of available items are provided in the table. The capacity of the knapsack is 7. (**b**) In the experiment, instances were presented on a computer screen. Each item was represented by a square. An item could be added to and removed from the knapsack by clicking on it. Selected items were displayed in green. (**c**) The state of the knapsack can be represented by an array of 0 s and 1 s with length equal to the number of items available. (**d**) Admissible states (possible compositions of the knapsack that do not violate the weight constraint) can be represented as vertices in a graph. The position of a vertex on the abscissa is determined by the value of the knapsack it represents, and the position on the ordinate is determined by the distance, in item space, of the vertex from the optimal solution, i.e., by the number of items that need to be removed and added in order to reach the optimal solution (CG; see Supplementary Methods 1.2).

**Figure 2:**
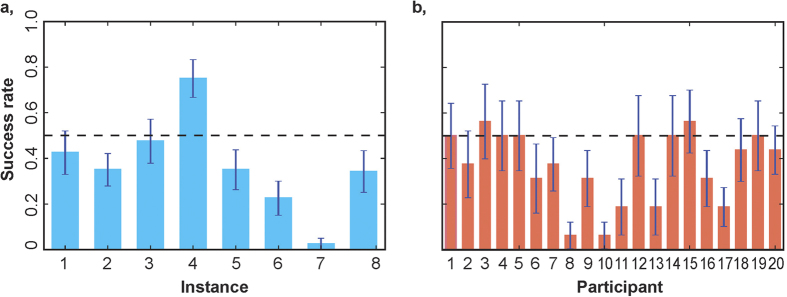
Variation in performance. (**a**) Success rates (proportion of successful attempts) for each of the eight instances administered in the task (see Table S1 for instance properties). (**b**) Success rates for each of the 20 participants. Blue lines indicate standard errors of means.

We examined whether participant behaviour in our task was consistent with key principles of decision theory. One such principle is that economic performance is related to effort extended. As effort increases, economic performance should too, that is, effort is *revealed* through (economic) performance. In the context of the KP, it is not immediately clear, however, how to define effort. For a computational device like a Turing machine, effort could be defined as the number of computational steps or running time of an algorithm that solves the problem. Since we cannot directly observe the number of computations performed by participants, we relied on proxies. Or first proxy is the length of the sequence of item additions to and removals from the knapsack in an attempt. These sequences can be mapped into a graph where vertices correspond to possible solutions, and edges indicate additions/removals of items (Fig. 1c–d, Fig. S1; Supplementary Methods 1.2). Path length then corresponds to the number of additions/removals (number of steps) required to travel from the initial vertex (empty knapsack) to a particular vertex. Terminal vertices in the graph correspond to capacity-constrained knapsacks: further additions would violate the weight constraint.

**Figure 3:**
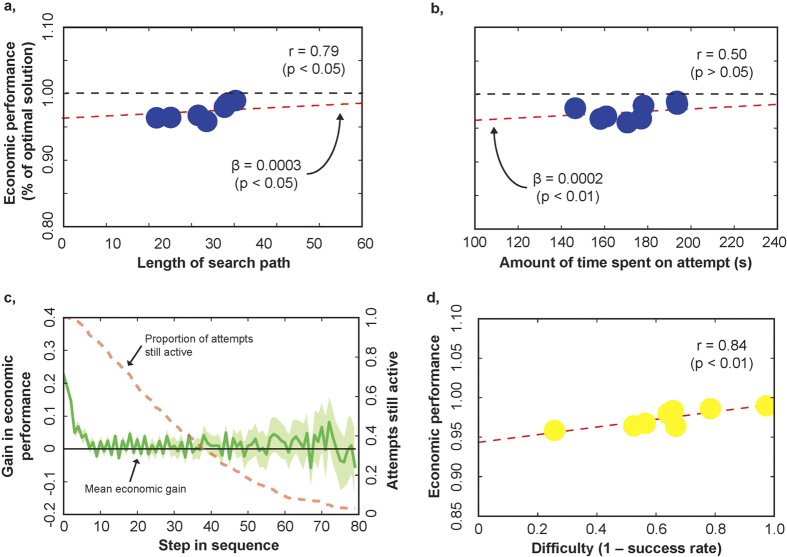
Economic Performance, Effort and Difficulty. (**a**) Scatter plot of economic performance (value attained as percentage of optimal solution value) and effort measured as mean length of the search path across attempts of an instance (instance means). Black dashed line: 100%, red dashed line: main effect of path length, linear mixed model (LMM) with random effects on intercept for individual participants (Table S3 Model 3), r: Pearson correlation, *β*: coefficient estimate of main effect. (**b**) Scatter plot of economic performance and effort measured as mean amount of clock time spent across attempts of an instance (instance means). Red dashed line: main effect of path length, LMM with random effects on intercept for individual participants (Table S3 Model 4). (**c**) Mean marginal gain in economic performance across all attempts, per step in the search path. (**d**) Scatter plot of economic performance against difficulty (as revealed by mean success rate across attempts of an instance; inverted scale) (instance means).

We first investigated whether path length measured human effort. According to decision theory, if path length is an appropriate measure of effort, economic performance should increase with path length. We found that economic performance increased in path length of attempts (*P < *0.05, main effect of path length, linear mixed model (LMM) with random effects on intercept for individual participants; Supplementary Results 2.3, Table S3 Model 3; Fig. 3a).

A related, alternative measure of effort is clock time. In principle, there was no opportunity cost to time in our experimental setting, because participants were barred from other activities while engaged in our task. Thus, clock time in itself cannot measure effort. However, clock time increases in the number of computations performed, as well as other dimensions of effort. The latter include differences in energy required across types of computations. These dimensions of effort are not recognised as effort in Turing machines, but may constitute effort for humans. As such, clock time may provide a catch-all measure of effort. Indeed, we found that time spent on solving an instance was related to higher economic performance (*P < *0.05, main effect of clock time, LMM with with random effects on intercept for individual participants; Supplementary Results 2.3, Table S3 Model 4; Fig. 3b).

In our task, earnings increased with a reduction in distance of value of submitted solution relative to optimal solution. As such, participants were not incentivised to find the optimal solution but only to get as close as possible to the optimal solution value. We therefore did not expect our measures of effort to correlate with computational performance, that is, whether the optimal solution was reached. Path length was indeed not related to computational performance (*P > *0.05, main effect of path length, generalised linear mixed model (GLMM) with random effects on intercept for participants; Supplementary Results 2.3, Table S3 Model 1); nor was time spent on an attempt (*P > *0.05, main effect of clock time, GLMM with random effects for participants on intercept, Supplementary Results 2.3, Table S3 Model 2).

### Difficulty and Economic Performance

A second key economic principle is related to marginal value of effort. A traditional economic agent (“Homo Economicus”) would search for a solution until marginal gain equaled marginal cost of effort. Measuring effort in terms of our ‘catch-all’ metric, clock time, marginal cost of effort must have been strictly positive because participants almost never exhausted the time limit in their attempts. With strictly positive marginal cost of effort, we therefore expected mean marginal gain to remain strictly positive throughout their effort spending. To this end, we plotted economic gain against time (Fig. S2a). Contrary to expectations, mean marginal gain quickly dropped to zero, and participants continued to search well beyond this point. As such, most of the search time was spent at zero marginal gain, violating one of the basic principles of economic theory. The same picture emerged when we plotted marginal gain in economic performance against path length, our other measure of effort (Fig. 3c).

Recently, the principle that economic agents work until marginal gain equals marginal cost, has become the subject of criticism^15^. An alternative proposal is that economic agents are boundedly rational and, due to their cognitive limitations, work only until they reach an aspiration point, which is less computationally demanding. Even if this was the case, in our setting economic gains should have remained positive until participants stopped spending effort, while they were not. One might rebut that our results could be explained by unrealistically high aspiration levels, and that participants therefore continued to spend effort even if economic performance did not increase. However, in this case, we would expect participants to spend effort until the end of allotted time, which they did in only 12 out of 320 attempts (see above).

Turning to difficulty, instance difficulty could be measured by success rate, that is, computational performance, which should *reveal* instance difficulty as experienced by our participants. One can reasonably posit that, as difficulty increases, marginal gain in economic performance decreases faster as effort increases. Because of this, we expected participants to have spent less effort, and hence, have attained lower economic performance, in instances where they were less successful. Contrary to this expectation, we actually found that economic performance increased with instance difficulty (Pearson correlation *r* = 0.838; *P < *0.01; Fig. 3d). This is rather paradoxical and raises a question: how could participants have sensed which instances were more difficult? To answer this question, we need to explore the nature of instance difficulty for humans, which we turn to in the next section.

The positive correlation between difficulty and economic performance could have been the result of the overall negative relation between path length from a solution to the optimum solution (*C*_*G*_, see Supplementary Methods 1.2), on the one hand, and solution value, on the other hand. To be specific, in our case, the average correlation between the values of the vertices and their distances from the optimum solution was *−*0.22 (min = *−*0.41; max = 0.04; Supplementary Methods 1.2). Because of this negative relation, solution attempts with higher economic performance (that is, with values closer to the optimum value) tended to be farther away from the optimum. Nevertheless, while computational performance was higher in instances where this negative relation was stronger, the correlation was insignificant (Pearson correlation *r* = *−*0.256; *P* > 0.05).

### Difficulty

In the next step, we investigated what constituted difficulty for participants. We wanted to determine whether difficulty was related to the notion of (computational) complexity as developed on the basis of idealised models of computation such as a Turing machine. However, we also went beyond those notions of complexity and investigated the link between difficulty and the potential for computational mistakes.

For a computational problem like the KP, the number of computations required to solve the problem in a mathematical model of computation such as a Turing machine, increases in the number of items. We found that computational performance indeed decreased in the number of items in an instance, that is, instances with more items were revealed to be more difficult (*P < *0.001, main effect of number of items, GLMM with random effects on intercept for individual participants; Supplementary Results 2.5, Table S4 Model 1). This is evidence for a relation between instance difficulty as experienced by humans, and as defined on the basis of a mathematical model of computation such as a Turing machine.

Computer science assigns computational problems to complexity classes, which are sets of functions that can be computed within given resource bounds^16^. Resource constraints of particular interest are time and memory. The problem of finding the solution to the 0–1 KP is a member of the complexity class non-deterministic polynomial-time hard. Membership of the complexity class is determined based on the worst instances of the problem. Other instances may require far less resources. To investigate the effect of computational complexity on human behaviour, we constructed measures that are related to the computational resources required by various computer algorithms designed for the 0–1 KP. We then related those measures to task performance.

In particular, we considered two classes of algorithms (Supplementary Methods 1.3, 1.6). One class of algorithms are *uninformed* search algorithms, which find the solution by exhaustive listing of elements in the search space^17^. The latter can be formalised as a graph and thus its size can be expressed as the number of vertices in this graph (Supplementary Methods 1.2). We found that difficulty experienced by humans increased with the total number of vertices in an instance’s graph (*P* < 0.001, main effect of number of vertices, GLMM with participant random effects on intercept; Supplementary Results 2.5, Table S4 Model 2), as well as with the number of terminal vertices (*P* < 0.01, main effect of number of terminal vertices, GLMM with participant random effects on intercept; Supplementary Results 2.5, Table S4 Model 3).

Another class of algorithms are *informed* search algorithms, which use a set of rules or heuristics to guide search^17^. The first algorithm we considered is based on dynamic programming^2^. It represents the KP in a way so that it can be solved by dynamic programming. The representation is a two-dimensional matrix of size equal to (number of items) × (base-2 logarithm of instance capacity), which is referred to here as *input size*. With this representation, a Turing machine with no memory constraints can solve the instance in polynomial time as a function of the size of the representation (Supplementary Methods 1.3, 1.5). However, this representation requires working memory larger than that of a typical human. For our first instance, for example, a Turing machine would need a memory of size 10 × log_2_ 1900 ≈ 109 bits, many times larger than the capacity of human working memory^18^. Therefore, we did not expect that humans would resort to dynamic programming. Consistent with this conjecture, the relation between input size and computational performance was not significant (*P* > 0.05, main effect of input size, GLMM with participant random effects on intercept; Supplementary Results 2.5, Table S4 Model 4).

**Figure 4:**
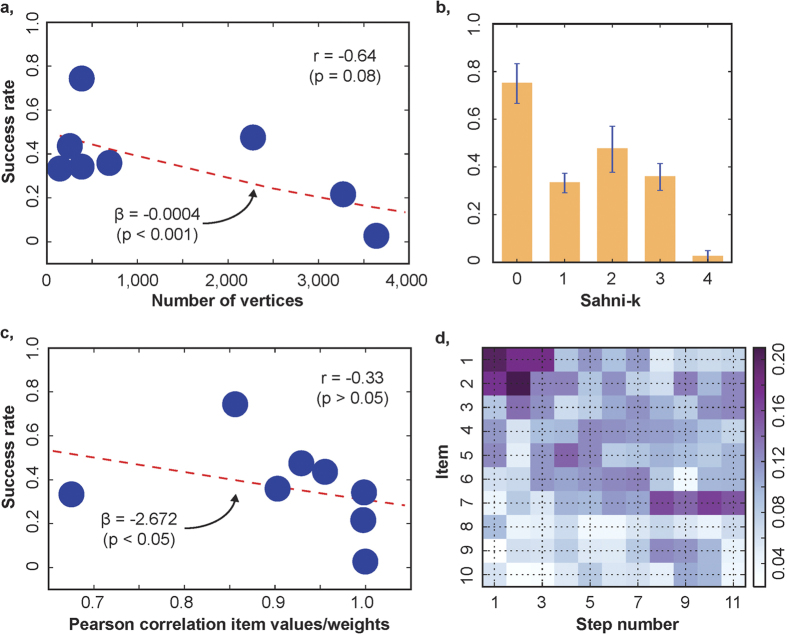
Properties of search. (**a**) Scatter plot of mean success rates across attempts of an instance and number of vertices in the graph of the instance. Red dashed line: main effect of number of vertices, general linear mixed model (GLMM) with random effects on intercept for individual participants (Table S4 Model 2), r: Pearson correlation, *β*: coefficient estimate of main effect. (**b**) Mean success rates across attempts of instances, stratified by Sahni-k level (Supplementary Methods 1.5). Blue lines: standard errors of means. (**c**) Scatter plot of mean success rates across attempts of an instance and Pearson correlation of item values and weights. Red dashed line: main effect of Pearson correlation between values and weights in instance, GLMM with random effects on intercept for individual participants (Table S4 Model 6), r: Pearson correlation, *β*: coefficient estimate of main effect. (**d**) Time course of choice frequencies of items. The items available in an instance were sorted in reverse order of their density (value-to-weight ratio; vertical axis). The heat map shows average choice frequencies for the items for the first 11 steps (horizontal axis) in the search path across all attempts.

Another important algorithm for the 0–1 KP is the Sahni-*k* algorithm^19^ (Supplementary Methods 1.3, 1.5). It solves an instance by a combination of brute-force search through a subset of items of cardinality *k*, and subsequently the greedy algorithm^20^. The greedy algorithm fills up the knapsack by picking items in reverse order of their value-to-weight ratio. If *k* equals zero, the Sahni-*k* algorithm coincides with the greedy algorithm. If *k* is equal to the number of items in the solution, the algorithm is similar to a brute-force search through the entire search space. *k* is proportional to the number of computational steps and the memory required to compute the solution of an instance. We define complexity of an instance as the minimum level of *k* necessary to solve the instance with the Sahni-*k* algorithm. If human approaches to the KP shared features with the Sahni-*k* algorithm, we would expect human-experienced difficulty to increase, and hence success rates to decrease, with *k*. Indeed, we found that computational performance was negatively correlated with Sahni-*k (P* < 0.001, main effect of Sahni-*k*, GLMM with participant random effects on intercept; Supplementary Results 2.5, Table S4 Model 5; Fig. 4b).

Many of the solution algorithms for the KP require arithmetic, which can be difficult for humans. For example, humans find it difficult to divide sequences of two numbers. Inaccuracies are more likely when numerators and denominators are highly correlated, that is, when the *conditioning number* is high^21^. In the greedy algorithm, items are ranked in descending order of their ratio of value over weight, and the knapsack is filled in this order until capacity is reached. The ranking will be less accurate if values and weights are more highly correlated. When correlation is perfect (as in our instance 7), ranking is impossible, and the greedy algorithm cannot be implemented. Therefore, if humans in part resort to applying the greedy algorithm, human-experienced difficulty should increase with, and economic performance should decrease with, the correlation between values and weights. We found indeed that computational performance was negatively related to this correlation (*P < *0.05, main effect of conditioning number, GLMM with participant random effects on intercept; Supplementary Results 2.5, Table S4 Model 6, Fig. 4c). However, the correlation did not explain differences in computational performance that Sahni-*k* could not capture (*P > *0.05, interaction effect Sahni-*k* × conditioning number, GLMM with participant random effects on intercept; Supplementary Results 2.5, Table S4 Model 7). This may have been the result of low power: with only 8 instances, insufficient independent variability is obtained across the Sahni-k metric and value-weight correlation.

### Solution Search Strategies

We found that economic performance decreased with Sahni-*k*, suggesting that participants’ search approaches combined the greedy algorithm with protocol that provided a disciplined, though not error-free, complement. This made us hypothesise that, at least in the early stages of solution attempts, our participants tended to use the greedy algorithm. Consistent with this hypothesis, we found that in the first few steps of their attempts, participants were more likely to add items with the highest value-to-weight ratio (Fig. 4d, Fig. S3). Since participants searched longer and with more success than the greedy algorithm (Supplementary Results 2.7), we conjectured that their complimentary protocol featured aspects of a branch-and-bound algorithm, where one starts with the greedy algorithm but then searches for improvements until a termination criterion is reached^22^ (Supplementary Methods 1.3). For high Sahni-*k* instances, where branch-and-bound algorithms deviate from from the greedy algorithm, we expected participants who were trying to replace multiple items at once, to realise a higher score. Indeed, with higher Sahni-*k*, only branch-and-bound algorithms that consider replacing several items at once can successfully find the optimal solution. We therefore tested whether individual differences in computational performance could be explained by the interaction between Sahni-*k* and the number of items replaced simultaneously between solution attempts. We measured the latter as the length of the shortest path between two full knapsack attempts, that is, between two terminal vertices in the instance graph (Supplementary Methods 1.2). The interaction term was indeed significant (*P < *0.01, interaction Sahni-*k* × mean distance, GLMM with random effect for instances on intercept; Supplementary Results 2.7, Table S6). As such, heterogeneity in search strategies across participants could be partly explained by how many items participants reconsidered and changed during their searches, and hence how complex the search protocol was as the distance between the greedy algorithm and the optimal solution increased.

**Figure 5:**
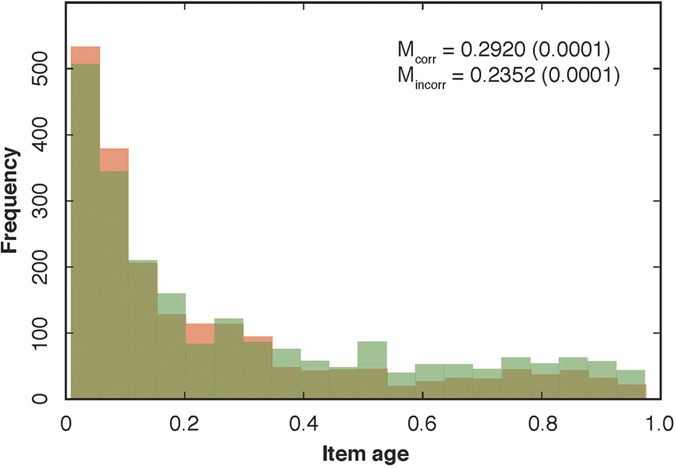
Distribution of item ages. Histogram of age of items (Supplementary Results 2.8). Age of an item was calculated as the fraction of number of steps taken since the beginning of an attempt (age equals 1 if the item was the first added to the knapsack). Green bars: correct items, red bars: incorrect items: *M*_*corr*_: mean age and standard error of mean of correct items, *M*_*incorr*_: mean age and standard error of mean of incorrect items.

Many algorithms depend on partial or complete memory of the search history in an attempt, for example, the highest value achieved so far and the items contained in the highest-valued knapsack. Here, too, humans may encounter difficulties, related to *episodic memory*. For example, some participants may not remember well why an item was put into the knapsack early on, and therefore decide to keep it in “because it must have been reasonable at the time.” We examined to what extent there was a tendency not to eliminate incorrect items that were added early on, and whether this determined computational performance. To this end, we considered the distribution of the age of incorrect items that were eventually deleted (Fig. 5). We measured age as the number of steps taken since the beginning of an attempt and until deletion, as a fraction of the total number of steps taken in an attempt; age equals 1 if the item was the first one to be added to the knapsack and removed only at the end of the attempt. The vast majority of deleted incorrect items were added very recently (M = 0.2920, SE = 0.0001); only rarely did participants eliminate incorrect items that were added to the knapsack early on. A similar pattern emerged for correct items that were deleted (M = 0.2352, SE = 0.0001; Fig. 5). This means that participants were reluctant to re-consider incorrect items that were added early on, introducing path dependence in search. Mean age of correct items was significantly higher than age of incorrect items (*P < *0.001, two-sample *t*-test, *t* = 6.98) and their distributions were significantly different (*P < *0.001, Kolmogorov-Smirnov test for independence of samples, *D* = 0.10). Direct evidence for path dependence emerged when we found that the initial full knapsack heavily determined eventual computational success: distance (path length) to the submitted solution from the optimum depended significantly on distance to optimum from the first terminal vertex (*P < *0.001, main effect of distance of first vertex, LMM with random effects for participants on intercept; Supplementary Results 2.8, Table S7 Model 1).

### Heterogeneity

We also examined variation in searches between participants. We found that there was little overlap in the knapsacks that participants attempted. On average, individuals only visited 3.6% of all vertices in the instance graph (Fig. S4a), yet combining all attempts, the twenty participants visited 42.1% of all vertices (Fig. S4b, Supplementary Results 2.6). This means that the group of 20 participants together explored more than fifteen times more of the search space than each individual participant explored on their own. This suggests that there was substantial heterogeneity in search strategies (Video S1–16). However, in all instances, at least one participant found the solution quickly (Fig. S5).

### Awareness

In the theory of computation, the problem of finding the solution of an instance, referred to as a optimisation version of the KP, is distinguished from the problem of deciding whether a given target value or greater is obtainable, referred to as the decision version. Given that the optimisation version of KP is NP-hard and the decision version is NP-complete, we wanted to determine whether participants who found the solution knew that they found the solution, and found it again in the second attempt. To test this, we examined their second attempts at the same instance. Among participants who found the solution in their first attempt, 31.2% did not solve the instance in the second attempt (Supplementary Results 2.9), which suggests that participants mostly were not aware that they had found the solution.

## Discussion

It has often been argued that humans resort to heuristics in order to solve complex problems^23^. Two questions have remained unanswered, however: (i) What type of complexity affects human decision-making? (ii) Are the heuristics humans use, adapted to this complexity? Here, we investigated how computational complexity affects human decision-making. We discovered that various measures of complexity explained computational performance, and hence, difficulty. Most prominently, the Sahni-*k* measure predicted success. This measure increases in the minimal number of items over which a combinatorial search has to be performed before the remainder of the knapsack can be filled using the greedy algorithm and the optimal solution can be attained.

This result replicates a finding of our earlier study^12^. In this study, 124 participants were asked to solve the same set of instances used in the present study. In this study, too, success rates were negatively correlated with Sahni-*k*^12^, suggesting that the reported relation between behaviour and computational complexity is robust.

The concept of computational complexity is defined with regards to mathematical models of computation, such as a Turing machine, an ideal model of computation. Our results demonstrate clearly that computational complexity, and hence the theory of computation, does help to understand when and how complexity impacts human decision-making. Our results were obtained in the context of the KP, a canonical example of a complex computational and decision problem, and future research should investigate to what extent computational complexity affects human behaviour in other problems.

Like for a Turing machine, we discovered that effort can be measured in terms of number of computations. However, we also found that this gives an incomplete picture. Our results indicate that problem-solving ability was limited by biological constraints including limited working and episodic memories as well as difficulty with arithmetic. Success rates decreased with Sahni-*k*, likely because more working memory is required for instances with higher Sahni-*k*. Working memory is known to vary between individuals; in our task, better working memory allows one to consider replacing multiple items at once, which is needed in order to be more successful at solving more difficult instances. Unwillingness to reconsider items selected many steps ago suggests that episodic memory constraints also affected behaviour. We further observed difficulties with division of numbers, required for successful implementation of the greedy algorithm. However, the number of instances participants were asked to solve was insufficient to disambiguate the influence of Sahni-*k* and division errors.

Despite memory and other computational constraints, participants scored well on our instances. We attribute this to algorithmic flexibility: humans appear not to stick to a single algorithm, but instead opportunistically change search procedures when encountering difficulties, for example, by deviating from the greedy algorithm when values and weights are highly correlated. Algorithmic flexibility was also evident in the surprisingly low overlap in composition of knapsacks attempted by the different participants.

To overcome memory and other computational constraints, humans could be provided with tools to facilitate solving the KP. A table akin to the one used in dynamic programming may resolve working memory problems. Likewise, forcing participants to re-consider items included early on in the search may help avoid the cognitive inflexibility caused by episodic memory. Explicit ranking of items by their value-to-weight ratios may facilitate the use of the greedy algorithm in the early stages of the search process.

One of our key findings is that participants tended to spend more effort on, and hence, improved economic performance in, more difficult problems. Relatedly, they tended to search way beyond the point where marginal economic gain decreased to zero. These results contradict basic principles of economic theory. Our results may explain important situations where humans do not trade off marginal utility against marginal effort, such as in labour-leisure choices. For instance, taxi drivers do not increase time spent on the job when marginal gains are high; instead, they increase effort per unit of economic gain on “difficult” days (days when passengers are harder to locate)^24^. An important question is how participants in our experiment were able to recognise that an instance was more difficult, because the measure that explained difficulty, Sahni-*k*, cannot be constructed until one knows the solution of an instance.

Our results cast doubt on a basic tenet of preference-based decision theory, namely, the assumption that decision-makers always optimise^25^. Decision theory assumes that agents will be able to identify the option they most prefer when faced with any finite, though potentially large, set of alternatives from the choice space. Our results suggest, however, that humans cannot find the optimum in many instances of an ubiquitous choice problem. Our results therefore cast doubt on the empirical relevance of modern preference-based decision theory. One real-world example of the KP is attention gating, which has been the subject of study in the emerging theory of optimal inattention^26^. There, economic agents choose optimally which items to attend to and which to ignore, trading off the benefits of attending to more items and the cost of doing so. It is questionable whether humans can execute the policies that emerge from such a preference-based theory of inattention. Our critique cannot be brushed aside by the argument that optimisation is merely “as if” (agents merely have to choose “as if” they optimise). The ability to represent choice in terms of optimisation rests on a basic axiom of preference theory, namely, completeness. If the optimisation problem is intractable, the preferences it represents are effectively incomplete. In general, we would advocate further development of a decision theory that takes seriously the computational demands involved^27^,^28^,^29^.

Across participants, we found little overlap in the knapsacks that they visited. This implies, first, that there does not seem to be a “representative heuristic” which captures the essence of all participants’ strategies. Second, it implies that information sharing has the potential to increase the number of participants that manage to solve a given instance. Markets are one way to indirectly share information, through prices. Our earlier study, involving 124 participants, indeed demonstrated that markets perform better than mechanisms such as prizes or patents that do not involve information sharing^12^. Our findings therefore have major implications for incentivising crucial human activities that can be thought of as solving instances of the KP, such as intellectual discovery and innovation^10^,^11^,^12^,^13^,^14^. There, recent advances have generally been attributed to the establishment of the patent system. But the patent system discourages information sharing, and as a result, could be improved upon through some information sharing mechanism. Or maybe this has already happened, because indeed, along with the introduction of patents, we have also witnessed a surge in the use of markets, which does lead to information sharing^12^. In any case, future research should more closely examine the relation between the KP and intellectual discovery and innovation.

We found that computational performance decreased with the size of the search space measured by the size of the graph of an instance. This result may potentially explain earlier findings that the number of decision options negatively affects choice, a phenomenon referred to as “choice overload”^30^. Our findings offer an algorithmic explanation of this phenomenon: the larger the number of choice options, the larger the search space through which the decision-maker has to search, and the less likely the decision-maker is able to discover the solution.

Interpretability of our results may be somewhat limited by the relatively small size of our sample (20 participants). Nevertheless, each participant solved 16 instances of the KP, resulting in 320 trials, and one of the main results, on the relation between participant success and a metric of difficulty from computer science, replicates the findings of an earlier study^12^ with 124 participants. This should suffice for the purpose at hand, which was to investigate average behavior.

Given the central importance of the KP in human life, we would advocate further investigation as a way to gain a more comprehensive understanding of human decision-making. This is bound to improve welfare not only through better incentivisation of, for example, intellectual discovery and innovation. It likewise promises to contribute to health through better diagnosis and treatment of mental disorders that implicate choice that is detrimental to successful KP solving, because of lack of cognitive flexibility (obsessive-compulsive disorder^31^), sub-optimal time allocation (attention deficit/hyperactivity syndrome^32^), or stringent adherence to “rational” economic principles (autism spectrum disorder^33^).

## Methods

### Participants and experimental task

Twenty human volunteers (age range = 18–30, mean age = 21.9, 10 female, 10 male), recruited from the general population, took part in the study. Inclusion criteria were based on age (minimum = 18 years, maximum = 35 years), right-handedness and normal or corrected-to-normal vision. The experimental protocol was approved by The University of Melbourne Human Research Ethics Committee (Ethics ID 1443290), and written informed consent was obtained from all participants prior to commencement of the experimental sessions. Experiments were performed in accordance with all relevant guidelines and regulations.

Participants were asked to solve eight instances of the 0–1 knapsack problem^2^. For each instance, participants had to select from a set of items of given values and weights, the subset of items with the highest total value, subject to a total weight constraint (Supplementary Tables, Table S1). The instances used in this study were used in a prior study^12^ and differed significantly in their computational complexity (Table S2).

The instances were displayed on a computer display (1000 × 720 pixels; Fig. 1b). Each item was represented by a square. Value and weight of an item were displayed at the centre of the square. The size of an item was proportional to its weight and the colour (share of blue) was proportional to its value. At the top of the screen, total value, total weight and weight constraint of the knapsack were displayed. When the mouse was moved over an item, a black frame around the square appeared and the counters at the top of the screen added this items’ value and weight to the totals. When the mouse was moved over an item that could not be added to the knapsack at that time, because its addition would have violated the weight constraint, the counters turned red. An item was selected into the knapsack by clicking on it. Once an item was selected into the knapsack, it turned green. The item stayed green until it was removed from the knapsack (by clicking on it again). A solution was submitted by pressing the space bar. An attempt was automatically terminated after 240 s and time remaining was displayed by a progress bar in the top-right corner of the screen.

Each participant had two attempts per instance. The order of instances was randomised across an experimental session. We recorded the time course of selection of items to and removals from the knapsack. To make the task incentive compatible, participants received a payment proportional to the values of their attempts (between $0 and $4 per attempt). In addition, participants received a show-up fee of $5.

### Data analysis

For each attempt, we recorded the sequence of additions of items to and removals of items from the knapsack. Each element in this sequence represents a state of the knapsack, and each state of the knapsack corresponds to a vertex in the graph *G* of the instance (the first element of the sequence always corresponds to the initial vertex of *G*, and the last element always corresponds to the participant’s proposed solution of the instance). A sequence of additions and removals can be represented as a path in the graph (see Supplementary Methods 1.2).

For each attempt, we recorded the time when the attempt was submitted as well as the sequence of additions and removals of items. For each step in this sequence, we computed the total value of items selected as well as the distance *C*_*G*_(*i, s*) to the solution vertex *s* from the vertex *i* in the graph representing this subset of items (see Supplementary Methods 1.2). The subset of items selected at the time of submission was the participant’s proposed solution of the instance. The attempt was marked correct if the subset of items in the participant’s proposed solution was the solution of the instance (that is, *C*_*G*_(*i, s*) was equal to zero), and incorrect otherwise.

To evaluate an attempt in value space, we computed the value of the proposed solution normalised by the value of the solution, on which the reward schedule depended. We also computed the difference between the proposed solution and the mean of the values of all terminal vertices in the graph representing the problem. The latter is the mean of the values of all maximally admissible knapsacks, which is equal to the expected value of randomly selecting items into the knapsack until the knapsack is full.

All analyses were performed in Python (version 2.7.6) and R (version 3.2.0).

## Additional Information

**How to cite this article**: Murawski, C. and Bossaerts, P. How Humans Solve Complex Problems: The Case of the Knapsack Problem. *Sci. Rep.* **6**, 34851; doi: 10.1038/srep34851 (2016).

## Supplementary Material

Supplementary Information
