# Supplementary material for: How Humans Solve Complex Problems: The Case of the Knapsack Problem
Source: Sci Rep. 2016 Oct 7;6:34851. doi: 10.1038/srep34851 (PMC5054396; doi:10.1038/srep34851)
Supplement: Supplementary Information [file srep34851-s1.pdf]

# How Humans Solve Complex Problems: The Case of the Knapsack Problem

Carsten Murawski[1] and Peter L.Bossaerts[1,2,3]

[1]Department of Finance, The University of Melbourne, Melbourne, Victoria 3010, Australia

[2]Florey Institute of Neuroscience and Mental Health, Melbourne, Victoria 3010, Australia

[3]David Eccles School of Business, University of Utah, Salt Lake City, UT 84112, USA

# SUPPLEMENTARY INFORMATION

## 1 Supplementary Methods

### 1.1 The 0-1 knapsack problem

The 0-1 knapsack problem is the problem of finding in a set of items of given values and weights the subset of items with the highest total value, subject to a total weight constraint [1, 2]. Mathematically, the problem can be written as

$$\max \sum_{i=1}^{I} v_i w_i \text{ subject to } \sum_{i=1}^{I} w_i x_i \leq C \text{ and } x_i \in \{0, 1\}, \tag{1}$$

where $I$ is the total number of items, $v_i$ and $w_i$, $i = 1, \ldots, I$, denote value and weight, respectively, of an item and $C$ is the capacity (maximum weight) of the knapsack.

The 0-1 knapsack problem is a combinatorial optimisation problem. Finding the optimal knapsack is a member of the complexity class *non-deterministic polynomial-time (NP) hard* and the corresponding decision problem of ascertaining whether a target value or greater can be obtained by a subset of items is a member of the complexity class *NP-complete* [2]. A complexity class is a set of functions that can be computed within given resource bounds [3]. Members of complexity classes differ in the rate at which computational resources, such as time and memory, grow as the size of a problem's instance increases. An important class comprises problems for which computational time increases as a polynomial of the problem's size (class polynomial-time, or P). If an algorithm exists that solves a problem in polynomial time, it is called "efficient" [4]. Thus, members of class P are those problems for which there exist efficient solution algorithms. The (optimisation version of the) KP is NP-hard, which means that there are no known efficient algorithms for it. Membership of a complexity class is determined based on the hardest instances of a problem and some instances of a given problem may require less time and memory than others.

## 1.2 Representing instances of the knapsack problem as graphs

An instance of the 0-1 knapsack problem can be represented as an undirected graph $G = (V, E)$ comprising vertices, $V$, and edges, $E$ [5]. We call a subset of items (knapsack) *admissible* if the combined weight of the items is less than or equal to the capacity of the knapsack, $C$. Each admissible subset $s$ of items is represented by a vertex $i \in V$. We define the *order* of a graph $|G|$ as the number of vertices of the graph. Note that $|G|$ will usually be lower than the number of all possible subsets of items, which is equal to $2^I + 1$ (including the empty set), because some possible subsets are not admissible due to the weight constraint. We define value $v_i$ and weight $w_i$ of a vertex $i$ as the sum of the values and weights, respectively, of the subset of items represented by vertex $i$. Two vertices $i, j \in V$ are connected by an edge $(i, j)$ if vertex $j$ can be reached from vertex $i$ by adding one item to or removing one item from the knapsack represented by vertex $i$, that is, if the difference between the sets of items represented by the two vertices contains exactly one item. Because the graph $G$ is undirected, $(i, j) = (j, i)$ for all $i, j \in V$. We call a vertex $i$ *incident* with edge $e$ if $i \in e$, and we call the two vertices $i, j \in G$ connected by edge $(i, j)$ *adjacent* to each other. We define the *degree* $d_G(i)$ of vertex $i$ as the number $|E(i)|$ of edges at $i$. We assign each edge $(i, j) \in E$ a weight $w_{ij}$ equal to 1. A *path* is a graph $P = (V', E')$ of the form $V = \{x_0, x_1, \ldots, x_k\}$ and $E = \{x_0x_1, x_1, x_2, \ldots, x_{k-1}x_k\}$, where the $x_i$ are all distinct. The vertices $x_0$ and $x_k$ are linked by $P$. We define the *length* of $P$ as the sum of the weights of its edges. We define the *distance* $\ell_G(i, j)$ in $G$ of two vertices $i, j$ as the length of a shortest $i$–$j$ path in $G$. The distance $\ell_G$ is conceptually related to the *edit distance* often used in computer science [6]. We call the graph $G$ representing a given instance of the 0-1 knapsack problem the *graph induced by the instance*.

We also define the undirected graph $\bar{G} = (\bar{V}, \bar{E})$ with vertices $\bar{V}$ and edges $\bar{E}$ defined as in $G$ except that edge weights are set equal to the value of the item whose addition to or removal from the knapsack is represented by the edge. Paths in $\bar{G}$ are defined similarly to paths in $G$. The distance $\ell_{\bar{G}}(i, j)$ of the two vertices $i$ and $j$ now represents the difference in values of the

two vertices. The solution of the knapsack problem represented by graph $\bar{G}$ can be found by computing the longest path in $\bar{G}$ [7]. We call the vertex $i \in V$ representing the solution of the instance the *solution node*.

Finally, we define the directed graph $\vec{G} = (\vec{V}, \vec{E})$ with vertices $\bar{V}$ as defined in $G$. Two vertices $i, j \in V$ are connected by an edge $(i, j)$ if vertex $j$ can be reached from vertex $i$ by adding one item to the knapsack represented by vertex $i$, and vice versa for removals of items. Note that in graph $\vec{G}$, $(i, j) \neq (j, i)$ for all $i, j \in \vec{V}$. We assign each edge $(i, j) \in \vec{E}$ a weight $w_{ij} = v_j - v_i$. Paths in $\vec{G}$ are defined as in $G$. The distance $\ell_{\bar{G}}(i, j)$ of the two vertices $i$ and $j$ represents the difference in values of the two vertices. We define the *out-degree* $d^{out}_{\vec{G}}(i)$ of vertex $i$ as the number of edges leaving vertex $i$, and the *in-degree* $d^{in}_{\vec{G}}(i)$ of vertex $i$ as the number of edges terminating at vertex $i$. We call the vertex in $\vec{G}$ representing the empty set (knapsack) the *initial vertex*. The initial vertex has an in-degree equal to zero. We call a vertex with out-degree equal to zero a *terminal vertex*. Each terminal vertex represents a *maximally admissible* knapsack, that is, a subset of items with the property that no additional remaining item could be added to the knapsack without violating the weight constraint. Note that the set of terminal vertices contains the vertex representing the solution of the knapsack problem. We consider all three graphs, $G$, $\bar{G}$ and $\vec{G}$, in the analysis of participants' attempts at solving knapsack problems.

Let us consider the graph $G = (V, E)$ of some instance of the 0-1 knapsack problem. The economic value of each node is given by $v_i$ for all $i \in V$. Let vertex $i$ represent the initial node (empty knapsack) and vertex $s$ represent the solution vertex. The distance between the two vertices $\ell_G(i, s)$ is equal to number of items in the solution of the instance. We can compute $\ell_G(i, s)$ for all other vertices $i \in G, i \neq s$. Intuitively, for any $i \in G, i \neq s$, $\ell_G(i, s)$ equals the number of additions of items to and removals of items from the knapsack to get from the knapsack represented by vertex $i$ to the solution of the instance, represented by vertex $s$. The mean correlation between vertex values $v$ and their distances to the solution vertex $\ell_G$ in the instances investigated in this study was $-0.22$ (min $= -0.41$, max $= 0.04$, SD $= 0.13$;

Tab. S2). Note that in convex problems this correlation would be positive. The low correlation between values and distances of vertices is one aspect of the knapsack problem that makes it hard. It means that optimisation algorithms based on local increase in marginal value such as hill-climbing do not work for the knapsack problem in general.

To illustrate this property, we plot the graphs of the instances investigated in this study in value-item (distance) space (Fig. S1). The position of each node in the graph of an instance is determined by its value (normalised by the value of the solution; abscissa) and distance (ordinate). The initial node is indicated in yellow and the solution node is indicated in red (top-right corner). As the plots illustrate, in each of the instances, there are many vertices of equal value but different distances, and vice versa.

### 1.3 Computational approaches to solving the 0-1 knapsack problem

Various algorithms have been proposed for the 0-1 knapsack problem. An algorithm is a tool for solving a well-specified computational problem [8]. It describes a specified computational procedure for achieving a desired relation between one set of values (input) and another set of values (output) that provides the solution to the computational problem. While every algorithm solves a particular computational problem, a given computational problem can often be solved by many different algorithms. This has led to the proposition that computational problems can be investigated separately from the algorithms that are used for solving these problems, that is, that the computational layer and the algorithmic layer are independent [9]. More recently, it has been suggested that computational and algorithmic layers are often interdependent and that therefore the study of the algorithmic layer can often provide important insights into the nature of the computational problem. This is also relevant for economics because the discipline has traditionally focused on the characterisation of the computational problems agents solve and ignored the way in which agents solve these problems. We propose that studying the search algorithms that humans may have used may give us important clues about the optimisation problem they were trying to solve.

Two classes of algorithms for the 0-1 knapsack problem can be distinguished: *uninformed* and *informed* search algorithms. Uninformed algorithms, such as breadth-first or depth-first search [10], typically search the entire graph of an instance to find the solution. Alternatively, the solution of an instance represented by graph $\bar{G}$ can also be found be computing the longest path in $\bar{G}$ [7]. Both running time and memory requirements of those algorithms increase non-polynomially in the size of the problem.

Informed search algorithms use some rule, sometimes referred to as *heuristic* [11], to guide the search. Instances of the 0-1 knapsack problem can be solved by dynamic programming [2]. Here, the time to compute the solution of a given instance is proportional to the *input size* of the problem given by $I \log_2 C$, where $I$ is the number of items in the instance and $C$ is the capacity (weight limit) of the knapsack. Running time and memory requirements of the dynamic programming algorithm still increases fast in the size of the problem and hence computation quickly becomes intractable.

Given the computational intractability of these approaches, various *approximation algorithms* have been developed for the knapsack problem. One important approximation algorithm is the *greedy algorithm* [8]. It solves the knapsack problem by selecting items according to decreasing *density* of the items, where density is defined as the ratio of value to weight of an item. The greedy algorithm has much lower computational demands than dynamic programming. It only requires sorting of the items. However, it is not guaranteed to find the solution of an instance and its proposed solution may be arbitrarily far away from the solution. Note that the greedy algorithm always finds the optimum in a variation of the instance in which fractions of items are allowed, that is, $w_i \in [0, 1]$, $i = 1, \ldots, I$ (LP-relaxation of the instance). The value of the solution in this modified problem (LP-bound) is often used as an approximation of the value of the solution in the 0-1 knapsack problem.

Another important type of algorithm is the *branch-and-bound* algorithm [12]. This algorithm starts with the greedy algorithm to construct an initial attempt and subsequently optimises the knapsack by selectively removing and adding items until a termination criterion has been

reached. Like the greedy algorithm, the branch-and-bound algorithm is not guaranteed to find the solutions of all instances of the 0-1 knapsack problem.

We also consider the *Sahni-$k$* algorithm [13]. The Sahni algorithm of order $k$ considers a subset of $k$ items and fills the knapsack using the greedy algorithm. It does so for all possible combinations of $k$ items. The proposed solution is the knapsack with the highest total value. Note that the Sahni algorithm of order zero is equal to the greedy algorithm. If $k$ is equal to the number of items in the solution of the instance, then the algorithm is equivalent to a brute force search and the solution proposed by the algorithm will be the solution of the instance. However, if $k$ is less than the number of items in the solution, then the Sahni algorithm is not guaranteed to find the solution of the instance.

### 1.4 Example instance of the 0-1 knapsack problem

We briefly discuss a small example instance of the 0-1 knapsack problem to illustrate the concepts discussed above. The properties of the instance are displayed in Fig. 1. There are five items available and the capacity of the knapsack is 7. Thus, the number of possible combinations of items is $2^5 = 32$ but only 18 combinations are feasible, that is, they meet the weight constraint. The solution is the set (1, 3) with total value 21 and weight 7.

At the bottom of the Fig. 1, the graph of the instance is displayed. The positions of vertices in the x-y plane are determined by their value (x-axis) and distance to the solution node (y-axis). The graph has 18 vertices, 31 edges connecting the vertices, and 7 terminal vertices (that is, maximally admissible sets). The empty set (initial vertex) is displayed in yellow and the solution vertex is displayed in red. The graph illustrates the low correlation between values and distance of the solution of the vertices. Some of the vertices have similar values but are at different distances to the solution, whereas other vertices have the same distance to the solution but different values.

As is typical of the 0-1 knapsack problem, some of the approximation algorithms would not find the solution of the instance. For example, the greedy algorithm would select items in

decreasing order of their value-to-weight ratio and end up with set (1, 2, 4) with total value 20 and weight 7.

## 1.5 Measuring difficulty of an instance

In computer science, problems are classified according to computational complexity. It is based on the resources required to solve the problem, irrespective of the algorithm used. The problem of finding the solution of the 0-1 knapsack problem is a member of the complexity class NP-hard [2]. We refer to this problem as the *search problem*. Membership of the class NP-hard means there is no known algorithm for finding solutions of instances (i.e., algorithms that solve the search problem) with the properties that the solution is correct and the running time of the algorithm is a polynomial of the instance's size (function of the instance's capacity and number of items). The members of class NP-hard are among the hardest computational problems currently known. The associated decision problem of determining whether a candidate solution is the optimal solution of an instance, is a member of the complexity class NP-complete [2]. Membership of a complexity class is based on the hardest instances of a problem, that is, instances of a given problem may vary in difficulty.

Difficulty can also be expressed in terms of various measures of the topology of the graph induced by an instance. They include the number of vertices in the graph representing the problem (number of admissible sets) and the number of terminal vertices in the graph (number of maximally admissible sets). These measures can be regarded as properties of the search space through which brute-force search algorithms have to search in order to find the solution, and computational time and memory requirements of most algorithms increase with the size of the search space.

The 0-1 knapsack problem is a special case of the class NP-hard because its instances can be solved by dynamic programming. The computational time of the dynamic programming algorithm for the 0-1 knapsack problem is proportional to $I \log_2 C$, where $I$ is the number of items available in the instance and $C$ is the capacity of the knapsack. Hence, the problem

is said to be *pseudo-polynomial*. We will refer to $I \log_2 C$ as the *input size* of the dynamic programming algorithm. It will be one of the measures of difficulty of instances.

Another measure of difficulty we consider here is based on the Sahni-$k$ algorithm described above [13, 14]. We define the $k$ of a given instance as the smallest order of the Sahni-$k$ algorithm that finds the exact solution of the instance. For example, an instance with $k = 0$ is an instance that can be solved with the Sahni algorithm of order 0, that is, the problem can be solved by applying the greedy algorithm. The higher the $k$ of an instance, the larger the distance of the instance's exact solution from the solution computed by the greedy solution. The higher the value of $k$, the higher are the number of computations and the memory requirement of the algorithm. The value of $k$ of the instances considered in the present study ranges from 0 to 4 (Tab. S2).

Another measure of difficulty of instances of the 0-1 knapsack problem is the Pearson correlation coefficient between values and weighs of the available items [15]. Stronger correlation is associated with greater difficulty. In many real-life situations, value and weight are strongly correlated. For example, in many investment problems, the return is proportional to the investment outlay plus a fixed charge for each project. If value and weight of items are strongly correlated, the instance is hard to solve for two reasons. Firstly, there is a large gap between the continuous (LP relaxation) and integer solution of the problem, and thus the problem is ill-conditioned. Secondly, sorting the items in decreasing order of their value-to-weight ratio means sorting according to their weights. This means, though, that for any small interval of the ordered items, there is only limited variation in weights, making it more difficult to satisfy the capacity constraint with equality [15].

Importantly, the measures of computational complexity described above are all defined relative to a Turing machine, a mathematical model of an idealised computing device. A Turing machine can perform a large number of computations, can have access to large amounts of memory and can perform mathematical operations with perfect accuracy. Thus, even real-world computers are not Turing machines, as their memory is limited and so is the precision

of mathematical operations. An important question is to which extent the measures of computational complexity transfer to humans. We expect that many of the properties of instances that make them hard for a Turing machine (or a real-world computer) also make them hard for humans. For example, the input size is proportional to the amount of memory required by the dynamic programming algorithm. Since human working memory is constrained, we would expect instances to become more difficult for humans as memory requirements increase, albeit at a different rate. Similarly, the Sahni-$k$ measure is a measure of the size of a combinatorial problem that needs to be solved in order to find the solution of an instance. For humans to solve combinatorial problems, they require working memory and they need to perform arithmetic. Thus, we expect that humans will perform worse on instances that require more memory, that is, instances with higher input size and higher Sahni-$k$ measures, *ceteris paribus*. On the other hand, in instances with low Sahni-$k$, most items are selected based on the greedy algorithm, which requires sorting according to the value-to-weight ratio. Since humans are prone to mathematical mistakes, we expect them to perform worse on instances with a high correlation between values and weights of items, *ceteris paribus*.

## 1.6 Participants and experimental task

Twenty human volunteers (age range = 18–30, mean age = 21.9, 10 female, 10 male), recruited from the general population, took part in the study. Inclusion criteria were based on age (minimum = 18 years, maximum = 35 years), right-handedness and normal or corrected-to-normal vision. The experimental protocol was approved by The University of Melbourne Human Research Ethics Committee (Ethics ID 1443290), and written informed consent was obtained from all participants prior to commencement of the experimental sessions.

Participants were asked to solve eight instances of the 0-1 knapsack problem [2]. For each instance, participants had to select from a set of items of given values and weights, the subset of items with the highest total value, subject to a total weight constraint (Table S1). The instances used in this study were used in a prior study [14] and differed significantly in their

computational complexity (Table S2).

The instances were displayed on a computer display (1000 x 720 pixels; Fig. 1b). Each item was represented by a square. Value and weight of an item were displayed at the centre of the square. The size of an item was proportional to its weight and the colour (share of blue) was proportional to its value. At the top of the screen, total value, total weight and weight constraint of the knapsack were displayed. When the mouse was moved over an item, a black frame around the square appeared and the counters at the top of the screen added this items' value and weight to the totals. When the mouse was moved over an item that could not be added to the knapsack at that time, because its addition would have violated the weight constraint, the counters turned red. An item was selected into the knapsack by clicking on it. Once an item was selected into the knapsack, it turned green. The item stayed green until it was removed from the knapsack (by clicking on it again). A solution was submitted by pressing the space bar. An attempt was automatically terminated after 240 s and time remaining was displayed by a progress bar in the top-right corner of the screen.

Each participant had two attempts per instance. The order of instances was randomised across an experimental session. We recorded the time course of selection of items to and removals from the knapsack. To make the task incentive compatible, participants received a payment proportional to the values of their attempts (between $0 and $4 per attempt). In addition, participants received a show-up fee of $5.

## 1.7 Data analysis

For each attempt, we recorded the sequence of additions of items to and removals of items from the knapsack. Each element in this sequence represents a state of the knapsack, and each state of the knapsack corresponds to a vertex in the graph $G$ of the instance (the first element of the sequence always corresponds to the initial vertex of $G$, and the last element always corresponds to the participant's proposed solution of the instance). A sequence of additions and removals can be represented as a path in the graph (Supplementary Methods 1.2).

For each attempt, we recorded the time when the attempt was submitted as well as the sequence of additions and removals of items. For each step in this sequence, we computed the total value of items selected as well as the distance $\ell_G(i, s)$ to the solution vertex $s$ from the vertex $i$ in the graph representing this subset of items (Supplementary Methods 1.2). The subset of items selected at the time of submission was the participant's proposed solution of the instance. The attempt was marked correct if the subset of items in the participant's proposed solution was the solution of the instance (that is, $\ell_G(i, s)$ was equal to zero), and incorrect otherwise.

To evaluate an attempt in value space, we computed the value of the proposed solution normalised by the value of the solution, which corresponds to the reward schedule. We also computed the difference between the proposed solution and the mean of the values of all terminal vertices in the graph representing the problem. The latter is the mean of the values of all maximally admissible knapsacks, which is equal to the expected value of randomly selecting items into the knapsack until the knapsack is full.

All analyses were performed in Python (version 2.7.6) and R (version 3.2.0).

# 2 Supplementary Results

## 2.1 Duration of attempts

In the following, we will only consider attempts that were submitted within the time limit of 240 s. Of all 320 attempts in the experiment, 12 were not submitted within the limit, leaving 308 attempts for analysis. The mean time spent on an attempt was 172.0 s (SD = 57.1). Means of instances (min = 146.5 s, M = 172.3 s, max = 193.7 s, SD = 15.7 s) were not significantly different (one-way ANOVA, $F(1, 6) = 5.2$, $P = 0.06$). We also fitted survival functions separately for each instance. We found that survival times differed significantly across instances (log-rank test, $\chi^2(7) = 14.9$, $P < 0.05$). Participant means (min = 73.4 s, M = 172.5 s, max = 226.7 s, SD = 39.8 s) were not significantly different from each other (one-way ANOVA,

$F(1, 18) = 0.13$, $P > 0.05$) but survival times differed significantly across participants (log-rank test, $\chi^2(19) = 303$, $P < 0.001$).

## 2.2 Quality of attempts

*Success rates:* The mean success rate, that is the proportion of attempts in which participants found the solution of an instance, was 37.4% (SD = 48.3%). In comparison, the expected success rate of an algorithm that fills knapsacks by picking items at random, which is equivalent to picking a maximally feasible knapsack at random, was 0.7%. The total number of successes was significantly above chance (one-sided binomial test, $P < 0.001$). The success rate varied substantially by both problem instance (min = 2.7%, M = 36.7%, max = 74.4%, SD = 19.3%; Fig. 2a) and participant (min = 6.2%, M = 37.4%, max = 56.2%, SD = 15.7%; Fig. 2b). One of the instances was only solved once and the participant who solved it had an overall success rate of 50.0% (there were 5 participants with higher average success rates). Note that performance varied more between problems (range = 71.7%) than between participants (range = 50.0%).

*Distance:* A refined measure of the quality of an attempt is the distance $\ell_G$ of an attempt from the solution in the graph $G$ induced by the instance (Supplementary Methods 1.2). The mean distance was 2.639 (SD = 2.325). It was significantly lower than the mean distance of attempts of an algorithm filling the knapsack by picking items at random, which was 5.068 (one-sample t-test, $t(307) = -18.374$, $P < 0.001$). Distance, too, varied significantly by both instance (min = 0.784, M = 2.622, max = 4.865, SD = 1.170) and participant (min = 1.429, M = 2.595, max = 4.062, SD = 0.765).

*Economic value:* To assess economic performance, we computed the value of a participant's attempt and normalised it by the value of the solution. Mean economic performance was 97.4% (SD = 5.8%). It was significantly higher than the expected economic performance of an algorithm that fills knapsacks by randomly picking items until the knapsack is full, which was 85.3% (one-sample t-test, $t(307) = 36.382$, $P < 0.001$). Similar to the previous performance measures, economic performance varied more by instance (min = 95.8%, M = 97.4%, max =

99.0%, SD = 1.1%) than by participant (min = 88.9%, M = 97.4%, max = 99.3%, SD = 2.4%).

A stricter benchmark to assess economic performance is the difference between the value of the solution of an instance and the expected value of a knapsack filled by randomly selecting items, normalised by the latter. It is a measure of economic performance relative to a random (skill-less) algorithm. The mean value of this shortfall measure was 79.7% (SD = 35.0%). This measure, too, varied significantly by both instance (min = 69.7%, M = 79.6%, max = 89.4%, SD = 6.2%) and participant (min = 36.4%, M = 79.8%, max = 94.3%, SD = 13.5%). The fact that this measure is significantly above 0 (one-sample t-test, $t(307) = 39.893$, $P < 0.001$) is another indication that human participants performed better than a skill-less (random) algorithm.

## 2.3 Effort and performance

Next, we examined the relation between effort and performance in more detail. One measure of effort spent on an instance is the number of additions of items to and removals from the knapsack, which we refer to as the length of the search path in the graph induced by the instance (Supplementary Methods 1.2). This number can be considered as a proxy of the number of computations performed by the participant during an attempt, that is, a measure of computational time (analogous to *CPU time* in computing). There was no relation between computational performance and path length ($P > 0.05$, main effect of path length, generalised linear mixed model (GLMM) with participant random effects on intercept and main effect of path length; Tab. S3 Model 1). We found a positive relation between path length and economic performance, measured as the value of an attempt normalised by the value of the optimal solution ($P < 0.05$, main effect of path length, linear mixed model (LMM) with participant random effects on intercept and main effect of path length; Tab. S3 Model 3). .

Another measure of effort spent on an instance is clock time. There was no relation between clock time spent on an instance and computational performance ($P > 0.05$, main effect of clock time, GLMM with instance and participant random effects on intercept and main effect of clock time, $P > 0.05$; Tab. S3 Model 2) but a positive relation between time spent on an

attempt and economic performance ($P < 0.05$, LMM with participant random effects on intercept and main effect of clock time; Tab. S3 Model 4). Participants who spent more time on an instance achieved higher values.

These results suggest that participants may have allocated resources (clock time and computational time on task) according to value. We investigated this notion in more detail. Homo economicus would be expected to keep spending effort on an attempt while marginal gain from effort is larger than marginal cost of effort. Thus, we would expect participants to keep working on an attempt as long as the marginal gain per unit of time is larger than the cost of effort (which we assumed to be positive and constant). To investigate whether this was the case, we computed marginal gain from effort per unit of clock time for each attempt and averaged across all attempts. We found that that mean marginal gain per unit of clock time dropped to zero at about 60 s and remained at zero for the remainder of time on task ( Fig. S2a). Given that the mean time on task was 172.0 s, as a group participants spent more than two thirds of their time on attempts at zero marginal gain. Indeed, if we assume that marginal cost of effort was strictly positive, as a group participants spent most of the time on task at a marginal net loss. The same pattern emerges when considering computational time instead of clock time (Figs. 3c and S2c).

We also examined how the quality of an attempt improved in item space. To this end, we computed the differences in distances $\ell_G$ to the solution between subsequent vertices in the path, which is equal to the gain in distance $\ell_G$ between two vertices, and examined the time course of gains. The mean gain reached zero after about seven steps (Fig. S2d) or about 70 s (Fig. S2b). This means that on average, the gains in quality of attempts were achieved in the first few steps of an attempt, after which the average gain was zero. We conclude that the gains in quality in attempts in both item and value space appeared in the first third to quarter of an attempt, after which gains in quality remained around zero on average.

In summary, more time spent on an attempt was associated with a higher economic performance in the attempt, but it was not associated with a higher computational performance. We now turn to the question of what determined computational performance.

## 2.4 Computational performance vs. economic performance

In the next step, we examined the relation between computational performance and economic performance. To this end, we compared success rates and economic values of attempts across instances. *Homo economicus* exerts effort until the marginal gain from effort is equal to the marginal cost of effort. The mean success rate can be interpreted as an index of difficulty of an instance. Assuming that marginal cost of effort is strictly positive and constant, we would expect a positive relation between computational performance and economic performance on average. That is, we would expect participants to make more money in instances with higher success rates (easy instances). However, we found the opposite to be the case: The mean value of attempts of an instance was negatively correlated with the mean success rate for the instance, that is, participants generated less value in easy instances compared to difficult instances (Pearson correlation $r = -0.838$, $P < 0.01$; Fig. 3d). This means that participants on average made more money on difficult instances. Note that for a given instance, correct attempts will always be worth more than incorrect attempts. The same applies for a given participant.

## 2.5 Variation in computational performance

We found significant variation in success rates (computational performance) across instances and also that success did not vary with time spent on those instances (Supplementary Results 2.3). We then investigated whether success in instances was related to instance properties, in particular various measures of their computational complexity and graph topology.

We first examined the relation between success and various measures of the size of the instances. Computational complexity is typically defined in terms of the size of an instance, which in case of the knapsack problem, is given by the number of items. We found that computational performance decreased in the number of items in an instance, that is, instances with more items were more difficult ($P < 0.001$, main effect of number of items, GLMM with with random effects on intercept for individual participants and main effect of number of items;

Tab. S4 Model 1). Computational performance was also negatively related to the number of vertices in the instance graph ($P < 0.001$, main GLMM with participant random effects on intercept and main effect of number of vertices; Tab. S4 Model 2, Fig. 4a). It was also negatively correlated with the number of terminal vertices at the level of individual attempts ($P < 0.01$, main effect of number of terminal vertices, GLMM with participant random effects on intercept with main effect of number of terminal vertices, Tab. S4 Model 3).

Next, we examined the relation between computational performance and computational complexity of the instance. Computational performance was not related to input size ($P > 0.05$, GLMM with participant random effects on intercept and main effect of input size; Tab. S4 Model 4). However, we found that computational performance was negatively related to Sahni-$k$ ($P < 0.001$, main effect of Sahni-$k$, GLMM with participant random effects on intercept and main effect of Sahni-$k$; Tab. S4 Model 5, Fig. 4b). The success rate of the instance with $k = 0$, that is, the instance that could be solved with the greedy algorithm, was 74.4% whereas the success rate for the instance with the highest $k$ ($k = 4$) was 2.7%. This suggests that there was a negative relation between computational complexity of the instances and success rate. We also found a negative relation between between computational performance and the Pearson correlation of item values and weights ($P < 0.05$, main effect of correlation between values and weights, GLMM with participant random effects on intercept and main effect of Pearson correlation between values and weights; Tab. S4 Model 6, Fig. 4c) but the value-weight correlation could not explain variation in performance that was not captured by Sahni-$k$ ($P > 0.05$, interaction Sahni-$k$ $\times$ Pearson correlation, GLMM with participant random effects on intercept, main effects for Sahni-$k$ and Pearson correlation between values and weights, and interaction Sahni-$k$ $\times$ Pearson correlation; Tab. S4 Model 7).

These results suggest that computational performance in the instances was strongly related to certain measures of the size of the search problem induced by the instance (size of the search space) as well as computational complexity of the instance. They provide indications of what search strategies or algorithms participants may have used and where their searches for solutions

broke down.

## 2.6 How did participants search?

To examine participants' search strategies in more detail, we considered the search paths during individual attempts, that is, the sequence of additions of items to and removal from the knapsack. From this sequence we can reconstruct the state of the knapsack at any point in time, which can be mapped on the instance graph as a search path. The average number of steps (item additions/removals) in participants' search paths was 33.3 (SD = 22.1). During their search, participants visited 4.0 terminal vertices (maximally admissible knapsacks) on average.

First, we computed the proportion of vertices and terminal vertices in the graph induced by an instance that participants visited during their search. The mean proportion of unique vertices visited by participants was 3.6%, with significant variation across instances (min = 0.6%, max = 7.6%, SD = 2.6%; Fig. 4a). As a group, they visited 42.1% of vertices of the instance graph on average (min = 10.2%, max = 74.8%, SD = 24.5%; Fig. 4b). This means that while individual participants only visited a very small proportion of the graph, as a group they visited a large part of it. This suggests that there was significant heterogeneity in search strategies. In addition, in all but one instance, at least one participant found the solution, which means that as a group, participants searched successfully whereas individually they did not. The mean proportion of vertices visited by participants was negatively correlated with the total number of vertices in the graph ($r = -0.888$, $P < 0.01$) and so was the proportion of vertices visited by the group ($r = -0.870$, $P < 0.01$).

We found a similar pattern for the proportion of unique terminal vertices visited by participants. The mean proportion of terminal vertices visited by participants was 4.6%, with significant variation across instances (min = 0.8%, max = 10.6%, SD = 3.1%; Fig. 4c). As a group, they visited 52.1% of terminal vertices on average (min = 12.5%, max = 74.0%, SD = 20.7%; Fig. 4d). There was also a large degree of heterogeneity in the number of terminal vertices submitted at the end of an attempt. The mean number of unique terminal vertices submitted by

participants was 13.9 (min = 7, max = 30, SD = 7.4). The mean proportion of terminal vertices visited by participants was negatively correlated with the total number of terminal vertices in the graph ($r = -0.861$, $P < 0.01$) and so was the proportion of vertices visited by the group ($r = -0.948$, $P < 0.001$).

We conclude that while individual participants only explored a relatively small part of the search space, as a group they explored a large part of it. Computational performance was not related to the proportion of vertices visited by participants ($P > 0.05$, main effect of proportion of vertices visited, GLMM with participant random effects on intercept and main effect of proportion of vertices visited; Tab. S5 Model 1) but it was positively related to the proportion of terminal vertices visited ($P < 0.05$, main effect of proportion of terminal vertices visited, GLMM with participant random effects on intercept and main effect of proportion of terminal vertices visited; Tab. S5 Model 2). That is, the extent of search had a small effect of computational performance but only with regards to terminal vertices.

We also investigated the relation between the extent of search and economic performance. There was no relation between economic performance and either the proportion of vertices or the proportion of terminal vertices visited ($P > 0.05$, main effects of proportion of (terminal) vertices visited, LMM with participant random effects on intercept and main effect of proportion of (terminal) vertices visited; Tab. S5 Models 3 and 4).

Next, we examined the *quality* of search. To do so, we compared the quality of the vertices visited to the average quality of the vertices in the graph. If participants picked vertices at random, then the quality of the vertices visited would be equal to the average quality of all vertices in the graph. First, we looked at the distance to the solution $\ell_G$ of vertices visited (Supplementary Methods 1.2). For each attempt, we computed $\ell_G$ of each of the vertices visited and computed the mean of those values. This gives us the mean of $\ell_G$ of all vertices visited. From it we subtracted the mean of $\ell_G$ of *all* vertices in the graph induced by the instance. The mean value of this difference was $-1.230$, which was significantly below zero (one-sample t-

test, $t(307) = -17.461$, $P < 0.001$). It implies that the quality of vertices visited by participants was significantly better than the average quality of vertices in the instances.

We found that the gains in quality of an attempt occurred mainly in the first stage of an attempt (Supplementary Results 2.3). To examine in more detail the notion that only the earlier but not the later stages of the search were beneficial, we considered the terminal vertices visited by participants during their attempts. More specifically, we compared the quality in item space of the first terminal vertex visited to the quality of the last terminal vertex visited. The first terminal vertex is the first full knapsack (set of items) a participant assembled and the last terminal vertex is the knapsack submitted. We measured quality of a vertex $i$ by its distance to the solution vertex $s$, $\ell_G(i, s)$ (Supplementary Methods 1.2). The mean distance of the first terminal vertex visited to the solution vertex across instances was 3.699 (min = 2.526, max = 5.350, SD = 0.876). In comparison, the mean number of items in the solution was 5.500 (min = 3, max = 9, SD = 1.871). The mean distance of the last terminal vertex was 2.628 (min = 0.763, max = 4.800, SD = 1.163). This means that there was a greater improvement in quality between initial vertex and first terminal vertex than between first and last terminal vertex visited. In addition, the mean difference between the terminal vertices visited, that is $\ell_G(i, j)$ where $i$ and $j$ are two subsequent terminal vertices on the search path, was 1.809 (min = 1.593, max = 2.052, SD = 0.131). Note that participants visited about 4 terminal vertices on average. This means that the mean distance between the terminal vertices visited was higher than the reduction in distances to the solution between first terminal vertex to last terminal vertex. It suggests that many of the changes in the sets of items between first and last terminal vertex did not result in a reduction of the distance to the solution.

We also computed the proportion of participants that had visited the solution vertex for each step in the search path. This gives us, for each step in the search, the proportion of participants who visited the solution vertex by that step. The mean proportion of participants across instances who visited the solution vertex was 39.6% (min = 2.7%, max = 79.5%, SD = 20.6%), which is slightly higher than the mean success rate. The mean number of steps across instances

until the first participant visited the solution vertex was 7.2 (min = 4, max = 12, SD = 2.9; Fig. S5), compared to a mean number of steps in the search path of 33.3. Across instances, among all participants who visited the solution vertex, the mean number of steps to the first visit was 18.0 (min = 8.6, max = 35.9, SD = 8.1). This means that among those participants who visited the solution vertex, the first participant to visit tended to be substantially faster than the average, another sign of heterogeneity in search strategies. However, most participants who visited the solution vertex kept searching before they submitted their solution. The mean number of steps between the first visit of the solution vertex and submission of the attempt was 21.1 (min = 6.1, max = 26.2, SD = 17.0). In this period, many participants visited the solution vertex multiple times before they submitted an attempt. The mean number of visits across instances, among those participants who visited the solution vertex at least once, was 2.8 (min = 2.0, max = 5.1, SD = 0.9). In addition, in some of the instances the proportion of participants who visited the solution vertex was higher than the success rate in the instances (Fig. S5). This suggests that some of the participants visited the solution vertex but submitted another set of items in the attempt, a point probably related to the NP completeness property of knapsack problems, which we examine in more detail below (Supplementary Results 2.9).

## 2.7 Which search algorithms did participants use?

Performance data in combination with information about the search path allows certain inferences about the type of search algorithm participants may have used. The relatively low computational performance together with the short average length of the search path and small fraction of the instance graphs participants explored, suggests that participants did not use any of the uninformed, exhaustive search algorithms.

On the other hand, participants' computational performance was substantially higher than that of a random algorithm, suggesting that participants used an informed (rule- or heuristic-based) algorithm. The fact that performance was well below 100%, rules out dynamic programming. This conclusion is further supported by the absence of a relation between success

rate and input size of the problem (Supplementary Results 2.5). It is more likely that participants used some sort of approximation algorithm. The finding that success rates decreased with the Sahni-$k$ of instances suggests that participants were using an algorithm of low computational complexity (Supplementary Results 2.5). The relatively high success rate in instances with a Sahni-$k$ of 0 suggests that the algorithm used was similar to the greedy algorithm. To investigate this possibility in more detail, we examined the sequence of additions of items to and removals of items from the knapsack. For each instance, we ordered the items in the various instances in decreasing order of value-to-weight ratio and computed the frequencies with which the items at each rank were chosen in the various steps of participants' sequences. If all participants used the greedy algorithm, then the items with the highest value-to-weight ratio of each instance would have been chosen in the first step, the item with the second highest value-to-weight ratio would have been chosen in the second step, and so on.

We found that across all participants and all instances, the items with the highest value-to-weight ratios were chosen most often in the first few steps. For example, the three items with the highest value-to-weight ratio were chosen in 15.6% of cases in the first three steps on average, while the mean frequency for the next seven items was 6.8% (Fig. 4d). In addition, the frequencies with which the items were chosen decreased with the number of steps. The three items with the highest value-to-weight ratios were chosen in 9.4% of cases in steps four to 10 on average, compared to 15.6% in the first three steps (Fig. 4d). These patterns were similar across all instances (Fig. S3). They suggest that participants selected the items with the highest value-to-weight ratios first when filling the knapsack, similar to the greedy algorithm. However, there was considerable variation in the order with which items were chosen, which suggests that participants either did not follow the greedy algorithm exactly or that not all participants followed the greedy algorithm.

Several other findings provide further support for the claim that participants did not use the greedy algorithm. Firstly, participants' performance was substantially higher than that of the greedy algorithm (it would only have found the solution in one of the instances whereas

participants solved 37.4% of instances on average). Secondly, the greedy algorithm fills the knapsack by selecting items into the knapsack in decreasing order of their value-to-weight ratio, until the knapsack is full. This means that the greedy algorithm would have terminated attempts after 6.6 steps on average. The average number of steps in participants' sequences (searches) was 33.3, however (SD = 22.1).

These results suggest that participants were more likely to have used an algorithm similar to branch-and-bound that starts the search by filling the knapsack with the greedy algorithm and then searches for improvements by systematically removing and adding items in search for higher value knapsacks. Since Sahni-$k$ is a measure of deviation of a solution from the greedy algorithm, we would expect that participants who tried to replace multiple items in the first full knapsack to be more successful, at least for instances were this was needed, that is, for high Sahni-$k$ instances. Therefore, we tested whether computational performance could be explained, not only by Sahni-$k$, but also by the interaction between Sahni-$k$ and the number of items participants replaced on average after reaching the first full knapsack. We measured the latter as the length of the shortest path between two full knapsack attempts, that is, between two subsequent terminal vertices (Supplementary Methods 1.2). The interaction term was indeed significant ($P < 0.01$, GLMM with random effect for participants on intercept, main effect of Sahni-$k$ and mean distance, and interaction of Sahni-$k$ $\times$ mean distance; Table S6). However, the fact that the values of the knapsacks did not increase over time for the last two thirds of participants' searches (Fig. S2a, S2c) suggests that participants did not use the branch-and-bound algorithm, at least not in its exact form. The high average length of sequences and the low average number of terminal vertices visited also suggests that participants did not use the Sahni algorithm.

## 2.8 Path dependence in search

Next, we investigated whether there was path dependence in the search paths. To this end, we examined the sequence of terminal vertices (maximally admissible knapsacks) visited by

participants during an attempt. First, we tested whether the distance $\ell_G(i, s)$ of the first terminal vertex $i$ to the solution $s$ was predictive of the distance of the last terminal vertex. We estimated a LMM with distance of the last terminal vertex as dependent variable and distance of the first terminal vertex as independent variable, with random effects on intercept for participants. We found that the distance of the first terminal vertex was predictive of the distance of the last terminal vertex, and hence of success ($P < 0.001$, main effect of distance of first terminal vertex, LMM with participant random effects on intercept and main effect of distance of first terminal vertex; Tabel S7 Model 1). This suggests that quality of an attempt (distance of the last terminal vertex to the solution) was path-dependent. We also found that the change in distances between first and last terminal vertex was predictive of the distance of the last terminal node ($P < 0.001$, main effect of change in distance, LMM with participant random effects on intercept and main effect of change in distance; Table S7 Model 2), which means that the quality of the search increased the likelihood of success.

We examined the notion of path dependence further by investigating to what extent there was a tendency not to eliminate incorrect items that were added early on, and whether this determined computational performance. To this end, we considered the distribution of the age of incorrect items that were eventually deleted (Fig. 5). We defined age as a fraction of number of steps taken since the beginning of an attempt (age equals 1 if the item was the first added to the knapsack). Most deleted incorrect items were added very recently (M = 0.2920, SE = 0.0001); only rarely did participants eliminate incorrect items that were added to the knapsack early on. A similar pattern emerged for correct items that were deleted (M = 0.2352, SE = 0.0001; Fig. 5). Mean age of correct items was significantly higher than age of incorrect items (two-sample $t$-test, $t = 6.98$, $P < 0.001$) and their distributions were significantly different (Kolmogorov-Smirnov test for independence of samples, $D = 0.10$, $P < 0.001$).

## 2.9 Did participants solve the decision problem?

In the theory of computation, a distinction is made between search problems and decision problems. The search problem is the problem of finding the optimal solution of an instance (also referred to as *optimisation problem*), whereas the decision problem is the problem of verifying that a candidate solution is the actual solution of an instance. In the case of the knapsack problem, the decision problem is 'Can a value of at least $V$ be achieved without exceeding the weight $C$?' The decision problem form of the knapsack problem is NP-complete, which implies that there is no known polynomial algorithm which can verify that the decision is true [2]. Given that the decision problem is NP-complete, the search problem of the knapsack problem is NP-hard, that is, there is no polynomial algorithm for solving the optimisation problem [16].

So far, we have analysed the search problem of the KP. We now examine whether those participants who submitted the correct solution, had actually solved the decision problem, that is, whether they knew that the candidate solution they submitted was the solution of the problem. As reported in the previous section, those participants who visited the solution vertex at least once tended to visit it several times. This suggests that those participants did not know that they had found the solution, that is, they could had not solve the decision problem. A participant who was able to solve the decision problem would have known that the solution vertex is indeed the highest value vertex, and hence would have submitted this set of items in their attempt. Moreover, considering only those attempts in which the participant visited the solution vertex at least once, in 6.5% of cases the participant subsequently submitted another set of items (of inferior value). These participants definitely did not solve the decision problem.

We also examined how participants performed on subsequent attempts of the same instance. Every participant attempted the same instance twice, with one attempt immediately following the other. A participant who solved the decision problem would be expected to remember the solution and therefore also solve the second attempt. Thus, we computed the number of times participants solved the first attempt of the same instance or the second or both. The

percentage of participant $\times$ instance pairs in which participants found the solution in at least one attempt was $51.0\%$. In $22.9\%$ of cases, participants solved both the first and the second attempt. In $17.6\%$ of cases, they only solved the second attempt, and in $10.4\%$ of cases they only solved the first attempt. Success in first and second attempt was not independent ($\chi^2$ test, $\chi^2(2, 153) = 23.3$, $P < 0.001$). We would expect the number of successful second attempts to be higher than the number of successful first attempts, as participants had already explored part of the search space. However, we would not expect there to be any cases in which a participant solved an instance in the first attempt but not in the second attempt. The fact that of all participants who solved instances at least once, $20.5\%$ only solved the instance in the first attempt but not in the second attempt, which indicates that those participants did not solve the decision problem, that is, they did not know that they had found the solution.

## Supplementary References


1. Mathews, G. B. On the partition of numbers. *Proceedings of the London Mathematical Society* **28,** 486–490 (1897).
2. Kellerer, H., Pferschy, U. & Pisinger, D. *Knapsack Problems* (Springer Science & Business Media, 2004).
3. Arora, S. & Barak, B. *Computational Complexity: A Modern Approach* (Cambridge University Press, Cambridge, 2010).
4. Fortnow, L. The status of the P versus NP problem. *Communications of the ACM* **52,** 78–86 (Sept. 2009).
5. Diestel, R. *Graph Theory* (Springer, 2006).
6. Navarro, G. A guided tour to approximate string matching. *ACM Computing Surveys* **33,** 31–88 (2001).
7. Dasgupta, S., Papadimitriou, C. & Vazirani, U. *Algorithms* (McGraw-Hill, New York, NY, 2006).
8. Cormen, T. H., Leiserson, C., Rivest, R. L. & Stein, C. *Introduction to Algorithms* (MIT Press, Cambridge, MA, 2001).
9. Marr, D. *Vision* (W.H. Freeman, San Francisco, 1982).
10. Russell, S. & Norvig, P. *Artificial Intelligence* (Pearson, Harlow, 2014).
11. Newell, A. & Simon, H. *Human Problem Solving* (Prentice-Hall, Englewood Cliffs, 1972).
12. Land, A. H. & Doig, A. An Automatic Method for Solving Discrete Optimization Problems. *Econometrica* **28,** 497–520 (1960).
13. Sahni, S. Approximate algorithms for the 0-1 knapsack problem. *Journal of the ACM* **22,** 115–124 (1975).
14. Meloso, D., Copic, J. & Bossaerts, P. Promoting intellectual discovery: patents versus markets. *Science* **323,** 1335–1339 (2009).
15. Pisinger, D. Where are the hard knapsack problems? *Computers & Operations Research* **32,** 2271–2284 (2004).
16. Moore, C. & Mertens, S. *The Nature of Computation* (Oxford University Press, Oxford, 2011).

# 3 Supplementary Figures

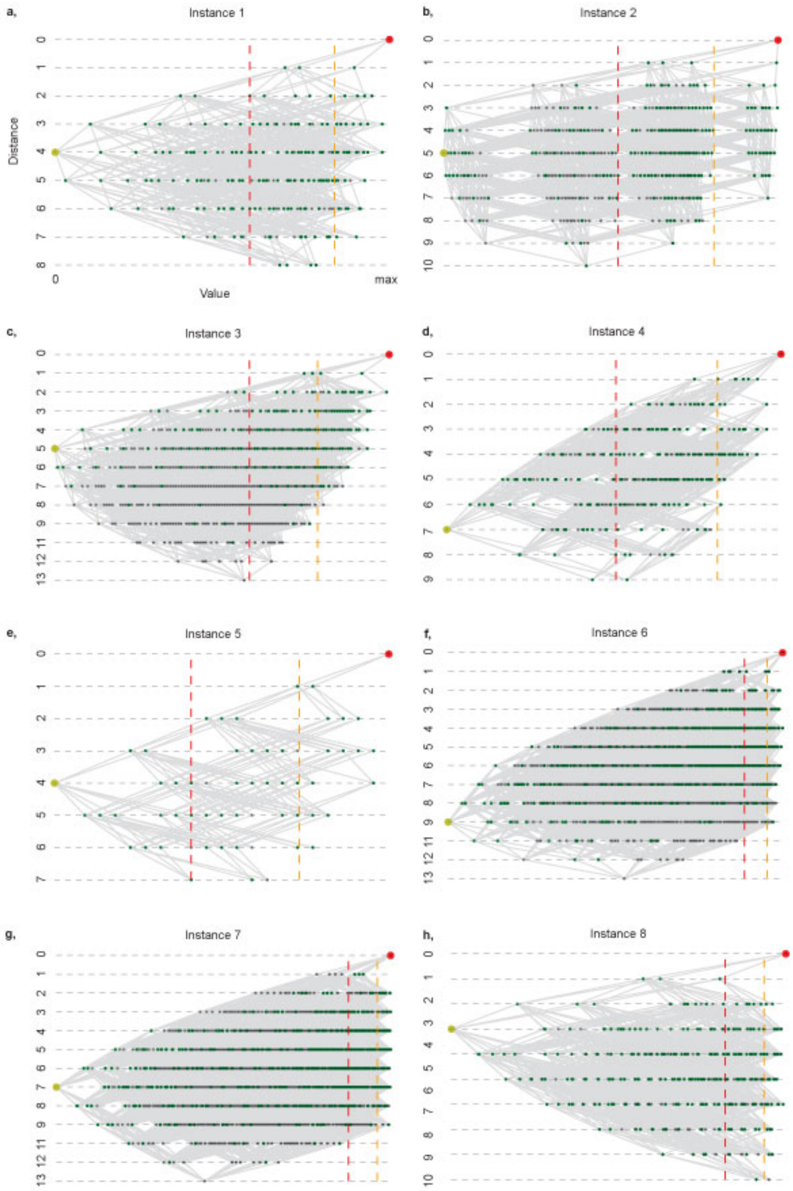


**Figure S1. Instance graphs for each instance in the task. a–h,** Graph induced by the instance (Supplementary Methods 1.2). Each vertex represents an admissable set of items. The initial vertex (empty set) is coloured in yellow and the solution vertex is coloured in red. Two vertices are connected by an edge if one vertex can be reached from the other by adding or removing one item. The position of a vertex on the abscissa is determined by the total value of the set of items represented by the vertex. The position of a vertex on the ordinate is determined by the distance $\ell_G$ (shortest path length) of the vertex to the solution vertex. The red dashed line indicates the lowest value of any terminal vertex and the yellow dashed line indicates the mean value of all terminal vertices. The vertices by participants during their attempts are coloured in green. The set of available items in each instance is provided in Table S1 and some key properties of the instance graphs are provided in Table S2.

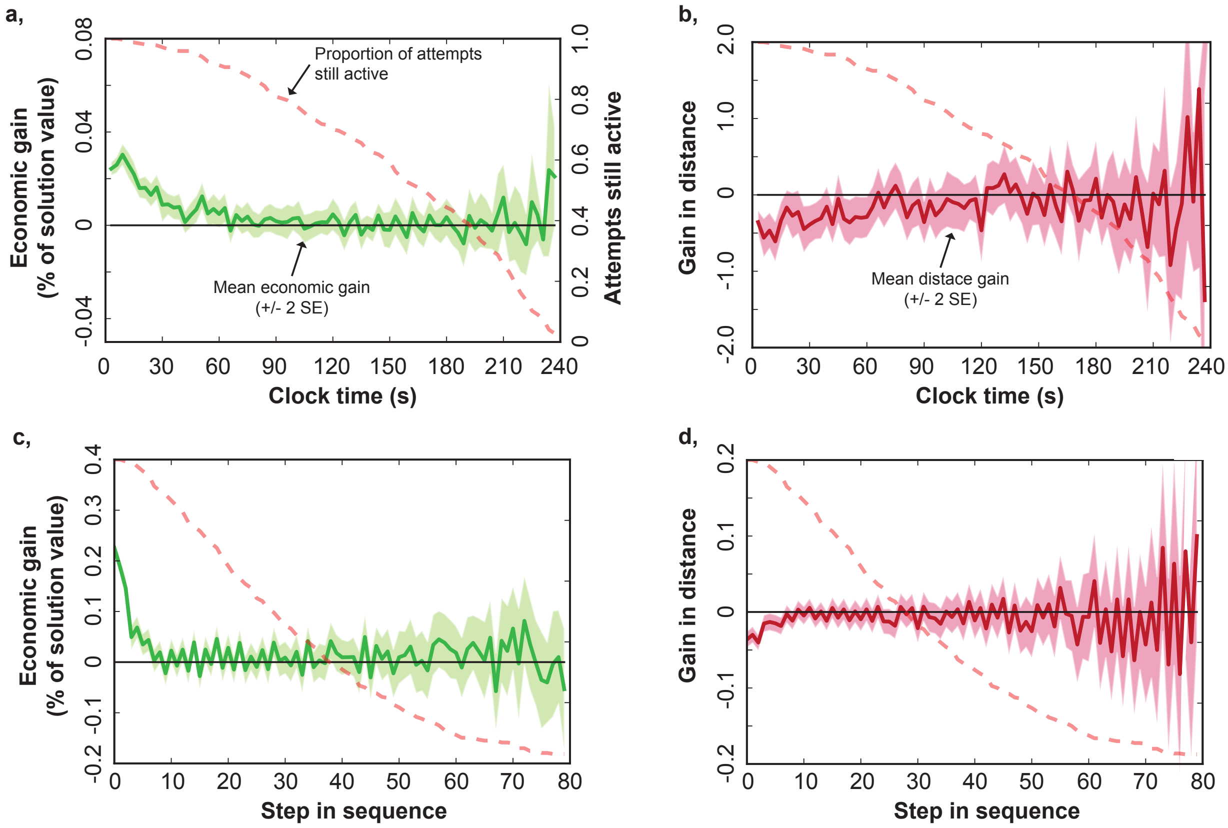


**Figure S2. Time courses of value gain and distance gain. a,** Time course of mean value gain per unit of clock time. The mean was computed over all attempts. **b,** Time course of distance gain per unit of clock time. The plot shows that mean change in distances $\ell_G$ per unit of clock time (Supplementary Methods 1.2). **c,** Time course of mean value gain per sequence step. **d,** Time course of distance gain per sequence step.

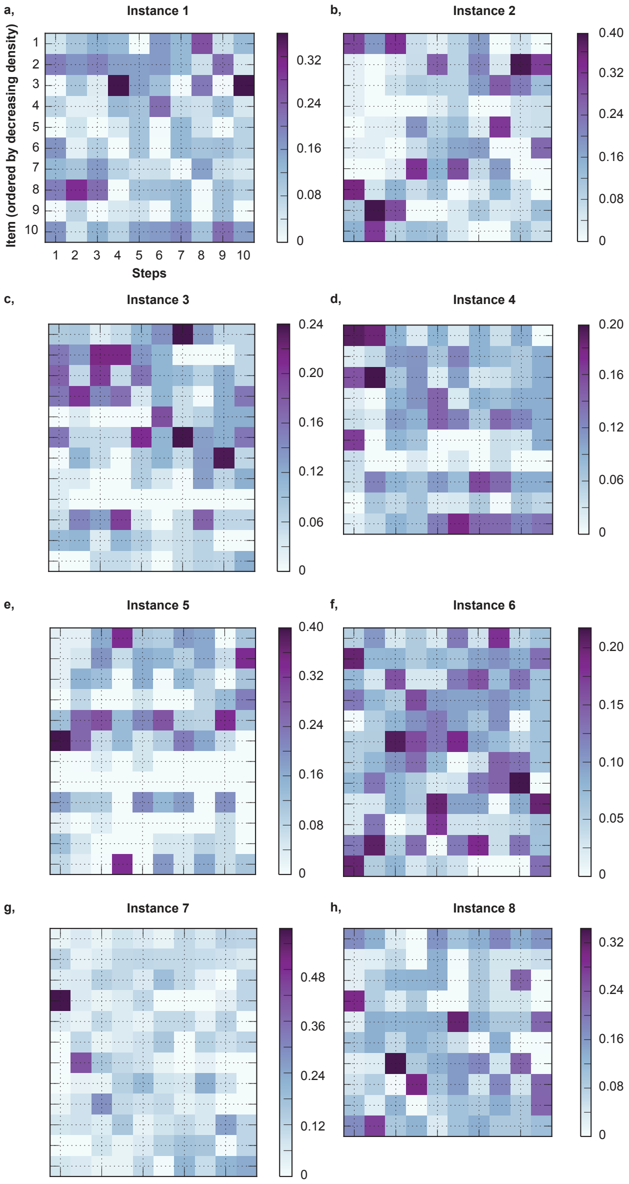


**Figure S3. Time courses of choice frequencies for individual items. a–h,** The items available in an instance were sorted in reverse order of their density (value-to-weight ratio). The heat map shows choice frequencies for the items for the first 11 steps in the search path (Supplementary Methods 1.2). If the greedy algorithm was used, off-diagonal entries would be zero.

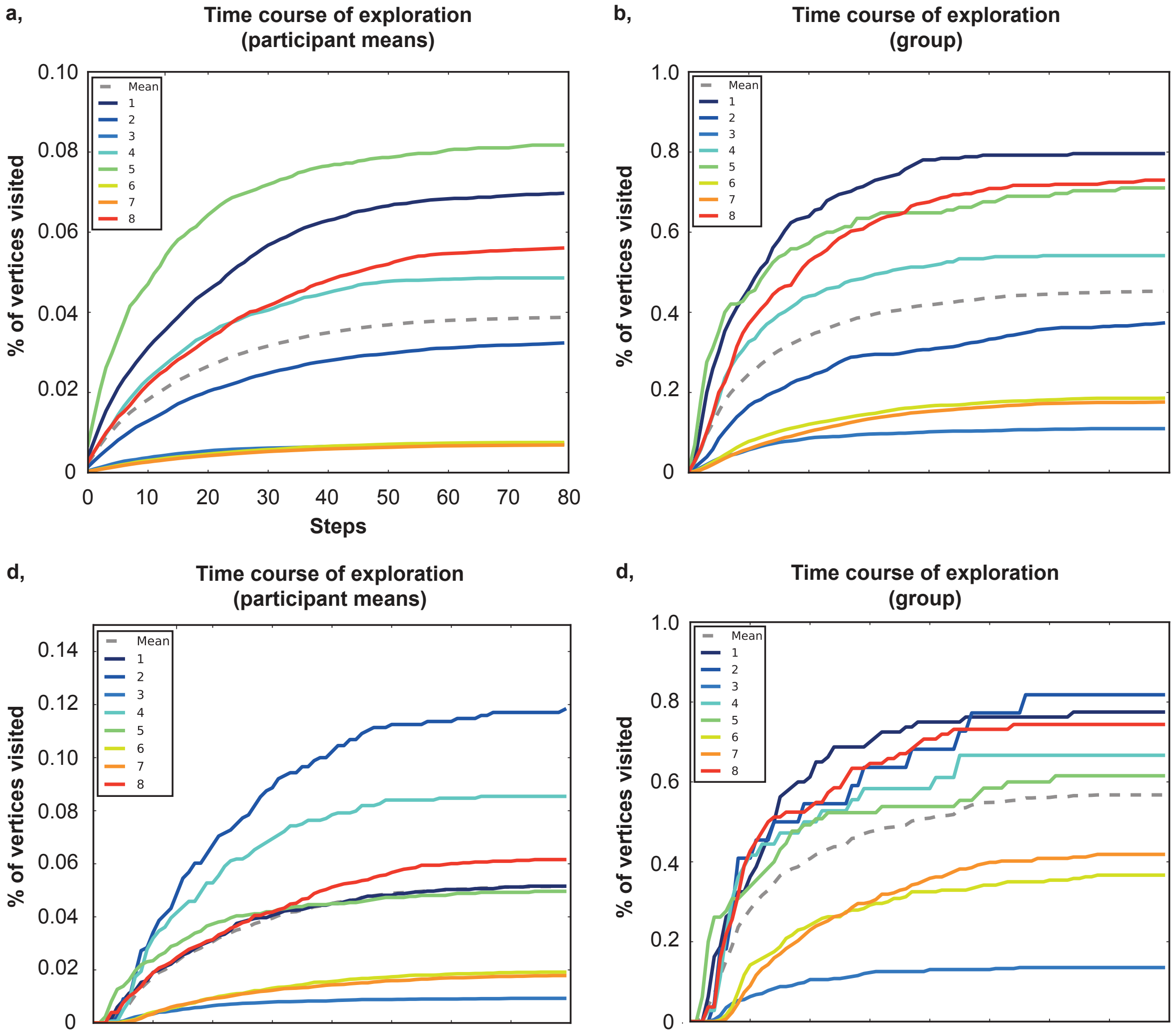


**Figure S4. Time courses of exploration. a,** Proportion of vertices visited by individual participants in each of the instances. The lines represent the mean of participant values in each of the instance. **b,** Proportion of vertices visited by all participants. The lines represent the proportion of vertices represented by the set of vertices visited by all participants at a particular step. **c,** Proportion of terminal vertices visited by individual participants in each of the instances. **d,** Proportion of terminal vertices visited by all participants.

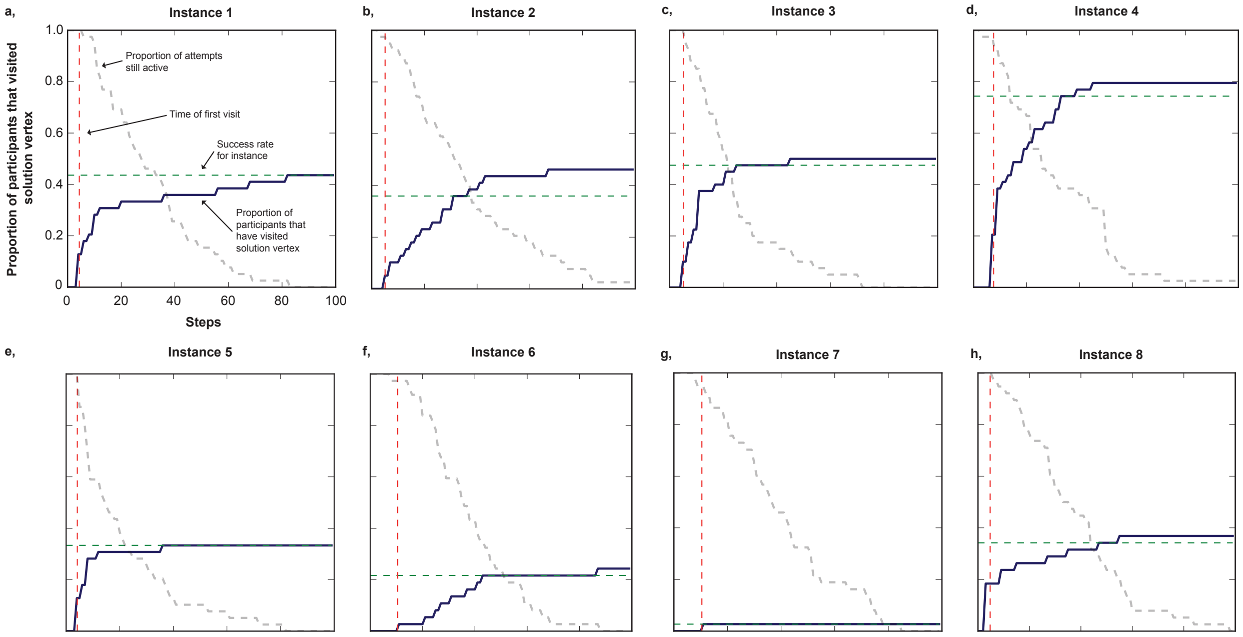


**Figure S5. Time courses of solution vertex visits. a–h,** The solid blue line shows the proportion of participants who have visited the solution vertex at a particular step during the attempt. The red dashed line indicates the step number at which the first participant visited the solution vertex. The green dashed line indicates the proportion of participants whose attempt was correct, that is, whose final set of items selected was identical to the set of items in the solution.

## 4 Supplementary Tables

**Table S1.** Available items and capacity (maximum weight) for each of the instances used in the experiment. Density is defined as the ratio of value to weight.

| **Instance 1** | Items | | | | | | | | | |
|---|---|---|---|---|---|---|---|---|---|---|
| *Capacity: 1,900* | 1 | 2 | 3 | 4 | 5 | 6 | 7 | 8 | 9 | 10 |
| Value | 500 | 350 | 505 | 505 | 640 | 435 | 465 | 50 | 220 | 170 |
| Weight | 750 | 406 | 564 | 595 | 803 | 489 | 641 | 177 | 330 | 252 |
| Density | 0.67 | 0.86 | 0.90 | 0.85 | 0.80 | 0.89 | 0.73 | 0.28 | 0.67 | 0.67 |

| **Instance 2** | Items | | | | | | | | | |
|---|---|---|---|---|---|---|---|---|---|---|
| *Capacity: 1,044* | 1 | 2 | 3 | 4 | 5 | 6 | 7 | 8 | 9 | 10 |
| Value | 300 | 350 | 400 | 450 | 47 | 20 | 8 | 70 | 5 | 5 |
| Weight | 205 | 252 | 352 | 447 | 114 | 50 | 28 | 251 | 19 | 20 |
| Density | 1.46 | 1.39 | 1.14 | 1.01 | 0.41 | 0.40 | 0.29 | 0.28 | 0.26 | 0.25 |

| **Instance 3** | Items | | | | | | | | | | | |
|---|---|---|---|---|---|---|---|---|---|---|---|---|
| *Capacity: 850* | 1 | 2 | 3 | 4 | 5 | 6 | 7 | 8 | 9 | 10 | 11 | 12 |
| Value | 15 | 14 | 3 | 3 | 10 | 9 | 28 | 28 | 31 | 25 | 24 | 1 |
| Weight | 129 | 144 | 77 | 77 | 66 | 60 | 184 | 184 | 229 | 184 | 219 | 72 |
| Density | 0.12 | 0.10 | 0.04 | 0.04 | 0.15 | 0.15 | 0.15 | 0.15 | 0.14 | 0.14 | 0.11 | 0.01 |

| **Instance 4** | Items | | | | | | | | | |
|---|---|---|---|---|---|---|---|---|---|---|
| *Capacity: 1,500* | 1 | 2 | 3 | 4 | 5 | 6 | 7 | 8 | 9 | 10 |
| Value | 37 | 72 | 106 | 32 | 45 | 71 | 23 | 44 | 85 | 62 |
| Weight | 50 | 820 | 700 | 46 | 220 | 530 | 107 | 180 | 435 | 360 |
| Density | 0.74 | 0.09 | 0.15 | 0.70 | 0.20 | 0.13 | 0.21 | 0.24 | 0.20 | 0.17 |

| **Instance 5** | Items | | | | | | | | | | | |
|---|---|---|---|---|---|---|---|---|---|---|---|---|
| *Capacity: 14* | 1 | 2 | 3 | 4 | 5 | 6 | 7 | 8 | 9 | 10 | 11 | 12 |
| Value | 2 | 3 | 4 | 5 | 6 | 9 | 8 | 7 | 6 | 5 | 8 | 9 |
| Weight | 3 | 4 | 6 | 3 | 5 | 13 | 6 | 9 | 2 | 4 | 7 | 7 |
| Density | 0.67 | 0.75 | 0.67 | 1.67 | 1.20 | 0.69 | 1.33 | 0.78 | 3.00 | 1.25 | 1.14 | 1.29 |

| **Instance 6** | Items | | | | | | | | | | | |
|---|---|---|---|---|---|---|---|---|---|---|---|---|
| *Capacity: 3,800* | 1 | 2 | 3 | 4 | 5 | 6 | 7 | 8 | 9 | 10 | 11 | 12 |
| Value | 107 | 35 | 120 | 206 | 88 | 34 | 28 | 110 | 88 | 101 | 74 | 53 |
| Weight | 599 | 196 | 670 | 1204 | 502 | 202 | 145 | 600 | 453 | 601 | 404 | 299 |
| Density | 0.18 | 0.18 | 0.18 | 0.17 | 0.18 | 0.17 | 0.19 | 0.18 | 0.19 | 0.17 | 0.18 | 0.18 |

| **Instance 7** | Items | | | | | | | | | | | |
|---|---|---|---|---|---|---|---|---|---|---|---|---|
| *Capacity: 1,300* | 1 | 2 | 3 | 4 | 5 | 6 | 7 | 8 | 9 | 10 | 11 | 12 |
| Value | 201 | 84 | 113 | 303 | 227 | 251 | 129 | 147 | 86 | 127 | 144 | 167 |
| Weight | 192 | 80 | 106 | 288 | 212 | 240 | 121 | 140 | 82 | 120 | 137 | 160 |
| Density | 1.05 | 1.05 | 1.07 | 1.05 | 1.07 | 1.05 | 1.07 | 1.05 | 1.05 | 1.06 | 1.05 | 1.04 |

| **Instance 8** | Items | | | | | | | | | |
|---|---|---|---|---|---|---|---|---|---|---|
| *Capacity: 265* | 1 | 2 | 3 | 4 | 5 | 6 | 7 | 8 | 9 | 10 |
| Value | 31 | 141 | 46 | 30 | 74 | 105 | 119 | 160 | 59 | 71 |
| Weight | 21 | 97 | 32 | 21 | 52 | 75 | 86 | 116 | 43 | 54 |
| Density | 1.48 | 1.45 | 1.44 | 1.43 | 1.42 | 1.40 | 1.38 | 1.38 | 1.37 | 1.31 |

**Table S2.** Properties of the instances of the 0-1 knapsack problem used in the experiment.

| **Instance** | **1** | **2** | **3** | **4** | **5** | **6** | **7** | **8** |
|---|---|---|---|---|---|---|---|---|
| Number of available items | 10 | 10 | 12 | 10 | 12 | 12 | 12 | 10 |
| Pearson correlation value/weight | 0.955 | 0.903 | 0.929 | 0.856 | 0.856 | 0.997 | 1.000 | 0.998 |
| Number of vertices in $G$ | 255 | 691 | 2,278 | 386 | 145 | 3,273 | 3,640 | 385 |
| Number of terminal vertices in $G$ | 80 | 22 | 399 | 36 | 65 | 240 | 301 | 82 |
| Number of edges in $G$ | 796 | 3,018 | 11,100 | 1,439 | 369 | 17,892 | 20,587 | 1,377 |
| Pearson correlation value/$\ell$ of vertices | -0.10 | -0.23 | -0.32 | -0.41 | -0.32 | -0.18 | -0.30 | 0.04 |
| Number of items in solution | 4 | 5 | 5 | 7 | 4 | 9 | 7 | 3 |
| Input size ($I \log_2 C$) | 109 | 100 | 117 | 105 | 46 | 143 | 124 | 80 |
| Sahni-$k$ | 1 | 3 | 2 | 0 | 1 | 1 | 4 | 3 |
| Success rate | 0.44 | 0.36 | 0.48 | 0.74 | 0.33 | 0.22 | 0.03 | 0.34 |
| Mean $\ell$ of attempt | 2.0 | 2.7 | 1.6 | 0.8 | 2.3 | 3.3 | 4.9 | 3.4 |
| Mean attempt value (% of solution value) | 0.97 | 0.98 | 0.96 | 0.96 | 0.96 | 0.99 | 0.99 | 0.98 |
| Mean length of search path | 31.6 | 37.7 | 25.1 | 33.6 | 21.8 | 38.6 | 40.3 | 38.2 |
| Mean % of vertices visited | 0.07 | 0.03 | 0.01 | 0.05 | 0.08 | 0.01 | 0.01 | 0.06 |
| % of vertices visited (group) | 0.80 | 0.39 | 0.11 | 0.54 | 0.71 | 0.19 | 0.18 | 0.17 |
| Mean % of terminal vertices visited | 0.05 | 0.12 | 0.01 | 0.09 | 0.05 | 0.02 | 0.02 | 0.06 |
| % of terminal vertices visited (group) | 0.78 | 0.82 | 0.14 | 0.67 | 0.62 | 0.37 | 0.42 | 0.74 |

**Table S3.** Relation between performance and effort at the level of individual attempts. Models (1) and (2) related the value achieved in an attempt, normalised by the value of the solution, to the length of the search path (1) and clock time (2). Models (3) and (4) related success in a single attempt to the the length of the search path (3) and clock time spent on the attempt (4). All models had random effects for participants on the intercept.

| | *Dependent variable:* | | | |
|---|---|---|---|---|
| | Attempt correct | | Value | |
| | *Generalized linear mixed-effects* | | *linear mixed-effects* | |
| | (1) | (2) | (3) | (4) |
| Length of search path | −0.005 (0.006) | | $0.0003^{*}$ (0.0002) | |
| Clock time on attempt | | −0.001 (0.003) | | $0.0002^{**}$ (0.0001) |
| Constant | −0.323 (0.502) | −0.389 (0.260) | $0.945^{***}$ (0.012) | $0.963^{***}$ (0.008) |
| Observations | 308 | 308 | 308 | 308 |
| Log Likelihood | −201.093 | −200.865 | 436.062 | 435.946 |
| Akaike Inf. Crit. | 408.185 | 407.730 | −864.124 | −863.891 |

*Note:* $^{*}p<0.05$; $^{**}p<0.01$; $^{***}p<0.001$

**Table S4.** Relation between performance and size and complexity of instances. Results of estimation of generalized linear mixed models relating success in an attempt to number of items in the instance (1), number of vertices in the instance graph (2), number of terminal vertices (3), input size (4), Sahni-$k$ (5), Pearson correlation between values and weights of items available in the instance (6) and factorial model with Sahni-$k$ and Pearson correlation between values and weights (7). All models had random effects for participants on the intercept.

| | *Dependent variable:* | | | | | | |
|---|---|---|---|---|---|---|---|
| | Success | | | | | | |
| | (1) | (2) | (3) | (4) | (5) | (6) | (7) |
| Number of items | −0.471*** (0.126) | | | | | | |
| Number of vertices | | −0.0004*** (0.0001) | | | | | |
| Number of terminal vertices | | | −0.003** (0.001) | | | | |
| Input size | | | | −0.005 (0.004) | | | |
| Sahni-$k$ | | | | | −0.514*** (0.108) | | 0.567 (1.817) |
| Pearson correlation value/weight | | | | | | −2.672* (1.169) | 1.183 (2.535) |
| sahni_k:item_corr_pearson | | | | | | | −1.138 (1.918) |
| Constant | 4.593*** (1.380) | −0.004 (0.207) | −0.176 (0.213) | −0.017 (0.475) | 0.343 (0.249) | 1.875 (1.073) | −0.755 (2.297) |
| Observations | 308 | 308 | 308 | 308 | 308 | 308 | 308 |
| Log Likelihood | −193.951 | −190.612 | −197.426 | −200.497 | −188.555 | −198.591 | −188.376 |
| Akaike Inf. Crit. | 393.902 | 387.224 | 400.853 | 406.993 | 383.110 | 403.181 | 386.752 |
| *Note:* | | | | | | | *p<0.05; **p<0.01; ***p<0.001 |

**Table S5.** Relation between performance and extent of search. Results of estimation of generalized linear mixed models relating success in an attempt to proportion of vertices in instance graph visited (1) and proportion of terminal vertices visited (2), and results of linear mixed model relating the value of an attempt, normalised by the value of the solution, to proportion of vertices in instance graph visited (3) and proportion of terminal vertices visited (4). All models had random effects for participants on the intercept.

| | *Dependent variable:* | | | |
|---|---|---|---|---|
| | Attempt correct | | Value of attempt | |
| | *Generalized linear mixed-effects* | | *Linear mixed-effects* | |
| | (1) | (2) | (3) | (4) |
| % of vertices visited | 4.853 (3.217) | | −0.052 (0.086) | |
| % of terminal vertices visited | | $5.102^{*}$ (2.302) | | 0.062 (0.060) |
| Constant | $-0.747^{***}$ (0.208) | $-0.827^{***}$ (0.205) | $0.976^{***}$ (0.006) | $0.971^{***}$ (0.006) |
| Observations | 308 | 308 | 308 | 308 |
| Log Likelihood | −200.080 | −198.662 | 440.411 | 440.408 |
| Akaike Inf. Crit. | 406.160 | 403.324 | −872.823 | −872.816 |
| *Note:* | | | $^{*}p<0.05$; $^{**}p<0.01$; $^{***}p<0.001$ | |

**Table S6.** Relation between computational performance and variation in search. Results of estimation of generalised linear mixed models relating success in an attempt to Sahni-$k$, the mean distance $\ell_G$ between subsequent terminal vertices visited during an attempt (Supplementary Methods 1.2) and the interaction between Sahni-$k$ and mean distance between subsequent terminal vertices. The model had random effects for participants on the intercept.

| | *Dependent variable:* |
|---|---|
| | *Attempt correct* |
| Sahni-$k$ | $-1.207^{***}$ (0.294) |
| Mean distance between terminal vertices | $-0.414$ (0.264) |
| Interaction Sahni-$k$ and mean distance | $0.354^{**}$ (0.134) |
| Constant | $1.115^{*}$ (0.531) |
| Observations | 279 |
| Log Likelihood | $-167.373$ |
| Akaike Inf. Crit. | 344.745 |
| *Note:* | $^{*}$p<0.05; $^{**}$p<0.01; $^{***}$p<0.001 |

**Table S7.** Path dependence in search. Results of estimation of linear mixed models relating distance $\ell_G$ between the last vertex in an attempt and the solution vertex (Supplementary Methods 1.2), to the distance of the first terminal vertex in an attempt from the solution vertex (1) and the mean change in distances between subsequent terminal vertices (2). The models had random effects for participants on the intercept.

| | *Dependent variable:* | |
|---|---|---|
| | Distance | |
| | (1) | (2) |
| Distance first to last terminal vertex | 0.560$^{***}$ (0.050) | |
| Change in distances | | 1.036$^{***}$ (0.055) |
| Constant | 0.566$^{*}$ (0.221) | 4.226$^{***}$ (0.134) |
| Observations | 304 | 304 |
| Log Likelihood | −638.128 | −571.910 |
| Akaike Inf. Crit. | 1,284.257 | 1,151.819 |
| *Note:* | $^{*}$p<0.05; $^{**}$p<0.01; $^{***}$p<0.001 | |
